# Single Nucleotide Polymorphism Array Profiling of Adrenocortical Tumors - Evidence for an Adenoma Carcinoma Sequence?

**DOI:** 10.1371/journal.pone.0073959

**Published:** 2013-09-16

**Authors:** Cristina L. Ronchi, Silviu Sbiera, Ellen Leich, Katharina Henzel, Andreas Rosenwald, Bruno Allolio, Martin Fassnacht

**Affiliations:** 1 Endocrine and Diabetes Unit, Department of Internal Medicine I, University Hospital, University of Würzburg, Wuerzburg, Germany; 2 Department of Internal Medicine IV, University Hospital, Ludwig-Maximilian-University Munich, Munich, Germany; 3 Institute of Pathology, University of Würzburg, Wuerzburg, Germany; Institut Jacques Monod, France

## Abstract

Adrenocortical tumors consist of benign adenomas and highly malignant carcinomas with a still incompletely understood pathogenesis. A total of 46 adrenocortical tumors (24 adenomas and 22 carcinomas) were investigated aiming to identify novel genes involved in adrenocortical tumorigenesis. High-resolution single nucleotide polymorphism arrays (Affymetrix) were used to detect copy number alterations (CNAs) and copy neutral losses of heterozygosity (cnLOH). Genomic clustering showed good separation between adenomas and carcinomas, with best partition including only chromosome 5, which was highly amplified in 17/22 malignant tumors. The malignant tumors had more relevant genomic aberrations than benign tumors, such as a higher median number of recurrent CNA (2631 *vs* 94), CNAs >100 Kb (62.5 *vs* 7) and CN losses (72.5 *vs* 5.5), and a higher percentage of samples with cnLOH (91% vs 29%). Within the carcinoma cohort, a precise genetic pattern (i.e. large gains at chr 5, 7, 12, and 19, and losses at chr 1, 2, 13, 17, and 22) was associated with a better prognosis (overall survival: 72.2 vs 35.4 months, *P=0.063*). Interestingly, >70% of gains frequent in beningn were also present in malignant tumors. Notch signaling was the most frequently involved pathway in both tumor entities. Finally, a CN gain at imprinted “IGF2” locus chr 11p15.5 appeared to be an early alteration in a multi-step tumor progression, followed by the loss of one or two alleles, associated with increased IGF2 expression, only in carcinomas. Our study serves as database for the identification of genes and pathways, such as Notch signaling, which could be involved in the pathogenesis of adrenocortical tumors. Using these data, we postulate an adenoma-carcinoma sequence for these tumors.

## Introduction

Adrenocortical tumors consist of highly prevalent adenomas (ACA) and rare carcinomas (ACC), which both can be either endocrinologically silent or hormonally active. ACC is a highly aggressive tumor with poor prognosis and an overall survival rate of 5 years of 25–50% in most series [1,2,3,4,5,6]. The rarity of ACCs is a major limitation to our understanding of their pathophysiology. Accordingly, the genetic mechanisms underlying adrenocortical tumor development are still largely unknown. In particular, it is still unclear whether ACCs evolve from ACAs following a second-hit paradigm. Although such a sequence has been occasionally observed [7], long-term follow-up of incidentally discovered adrenocortical neoplasms suggests that ACAs generally maintain a benign phenotype [8,9]. In addition, a recent study described a mouse model in which dysregulation of one pathway (i.e. β-catenin accumulation or IGF2 overexpression) may result in adrenal hyperplasia, but a second hit or multiple alterations appear to be necessary for tumorigenesis [10]. Moreover, distinguishing malignant from benign tumors remains a clinical dilemma as only the combination of several markers allows a clear-cut histological differentiation [11]. Thus, the diagnosis of ACC still requires histological examination and scoring by a highly experienced pathologist. The profound differences in treatment, follow-up and prognosis between benign and malignant adrenal lesions make the correct classification of adrenocortical tumors of utmost importance.

Molecular studies of inherited syndromes with increased risk of ACC, coupled with recent advances in expression profiling, have improved our understanding of the genetic mechanisms underlying ACC development [12,13,14,15]. For instance, microsatellite markers revealed common inactivating mutations or loss of heterozygosity (LOH) at 17p13, a region that includes the *TP53* tumor suppressor gene, LOH at 11q13 (*MEN1* locus), and alterations of the imprinted 11p15 locus (paternal isodisomy) leading to IGF2 overexpression [16,17]. Finally, constitutive activation of β-catenin is a common alteration in both ACA and ACC, being likely involved in both adrenocortical development and neoplasia [18].

Copy number alterations (CNAs) have also been described in adrenal hyperplasia, ACA and ACC using fluorescence in situ hybridization (FISH), karyotyping, and either conventional comparative genomic hybridization (CGH) [19,20,21,22,23,24,25] or array-based CGH [23,26,27]. In particular, losses at 1p, 2q, 3p, 6q, 9, 11, 17p, and 18q and gains at 4, 5, 12q, 16, 19 and 20q have been reported in patients with sporadic ACC, some of them being associated with a poor prognosis [28]. Taken together, gene expression and CGH studies have shown that several pathways are dysregulated in ACC affecting cell cycle, retinoic acid signaling, lipid metabolism, complement system, and antigen presentation [13,28,29]. However, results from CGH studies have been variable and restricted to large regions not allowing the identification of candidate genes associated with disease initiation or progression. Of note, most of the molecular candidates so far proposed as diagnostic or prognostic markers, like ERCC1 [30], GLUT1 [31] and MMP2 [32] have not yet been confirmed in subsequent studies, with the significant exception of the steroidogenic factor 1 [33,34].

In contrast to CGH, the single nucleotide polymorphism (SNP) microarray technique allows detection of both genome-wide CN data and LOH events [35]. By merging these two analyses, it is possible to identify copy neutral LOH (cnLOH, or uniparental disomy, UPD), a chromosomal defect which comprises 50-70% of all LOH events detected in human tumors [36]. Furthermore, recent high resolution SNP array platforms can identify amplifications/deletions at a single gene level, offering a powerful method for oncogene and tumor suppressor gene discovery [37,38,39]. Recently, we applied this method to investigate the genetic events in a group of 15 benign cortisol-secreting tumors, detecting several recurrent CN gains and losses, mostly microalterations, in both already known and newly identified chromosomal regions. In addition, we reported novel genes (i.e. *HRAS, EPHA7*, and *SGK1*) and pathways (i.e. Notch signalling pathway) that could be involved in early tumorigenesis [40]. Another recent study on childhood adrenocortical tumors also using SNP array profiling identified some amplified oncogenes and deleted tumor suppressor genes and demonstrated different oncogenic routes [41].

In the present study, we used high-resolution SNP microarrays to investigate a larger series of benign and malignant adrenocortical tumors with the major aim to identify new candidate genes that could be suggestive for an adenoma-carcinoma sequence (i.e. alterations common between ACA and ACC). Furthermore, we also intended to recognize new potential markers of malignancy or prognostic factors for ACC.

## Material and Methods

### Ethic statement

The study was approved by the ethics committee of the University of Würzburg (permits No. 93/02 and 88/11) and written informed consent was obtained from all patients.

### Study population

A total of 50 adrenocortical tumors with matched blood samples were selected for the present study independent of the hormonal status (26 samples from ACC patients and 24 samples from ACA patients). Clinical parameters, such as sex, age at diagnosis, date of surgery, tumor size, pathological classification, and results of hormone analysis were collected from patient records. Malignancy was defined according to established clinical, biochemical, and morphological criteria [42], while the biochemical diagnosis of cortisol hypersecretion was made according to established guidelines [43]. For ACC patients, additional data, such as tumor stage according to the ENSAT (European Network for the Study of Adrenocortical Tumors) classification, Weiss score, MIB1 (Ki67 index), presence and number of distant metastasis, follow-up duration, overall and disease free survival, type and response to treatment, was collected from the German ACC Registry [30,44].

### Tissue samples and DNA extraction

Tumor tissue specimens collected from patients operated between 1993 and 2011 were used for the present study. Corresponding blood samples were collected from all patients. Normal adrenal tissue surrounding the tumor was also available for 17 out of 53 patients. Tumor, blood and normal adrenal samples were stored at -80°C in our department (University Hospital, Wuerzburg, Germany). Genomic DNA was extracted using standard procedures (QIAamp DNA Mini and Blood Mini Kit, Qiagen, Hilden, Germany). Only samples with appropriate DNA quality as measured by Bioanalyzer 2100 (Agilent Technologies, San Jose, CA, USA) were further evaluated.

### SNP arrays and data analysis

SNP array experiments were performed using the high-resolution Affymetrix GeneChip Human Mapping 6.0 microarray (SNP 6.0, Affymetrix Inc., Santa Clara, CA, USA), as previously reported [40]. Affymetrix raw intensity data (.CEL files) were generated by the command console and quality control (QC) was assessed by the Genotyping Console software (GTYPE version 4.0, Affymetrix, Santa Clara, CA). Only arrays were accepted that had a QC of 1.7 or higher and a MAPD of 0.3 or smaller, as recommended for large study sets.

The CN analysis was performed using the Partek Genomics Suite software package (Partek GS, Version 6.5, Partek Incorporated, St. Louis, MI, USA, www.partek.com), as previously published [38,40]. A preliminary unpaired data analysis was performed in all samples (50 tumors, 50 blood samples and 17 normal adrenal tissues surrounding the tumors). Unexpectedly and for no obvious reasons, 8 blood samples (16% of total) showed a large number of CNAs. Similar results were obtained after repeated blood sampling, hybridization and data analysis. In four of these 8 cases, the tissue surrounding the tumor was available and was taken as normal reference, while the remaining 4 samples were excluded from further analysis. The paired CNA analysis was carried out by comparing the intensity of the hybridization signal from a tumor sample to that of the matched normal DNA. The genomic clustering was performed by unsupervised euclidean distance clustering (complete linkage), while regions of CNA were detected by a genomic segmentation algorithm, as previously described [40]. A false-discovery-rate based adjustment was always applied using the step up method with an alpha value of 0.05 (Benjamini and Hochberg, 1995).

Genotype analysis was performed using the Birdseed v2 algorithm (Affymetrix GTC, version 4.0), according to the recommended instructions. The SNP call rate after genotype analysis was >95% in all samples. For the LOH analysis, CHP files were imported into the Partek GS and the hidden Markov model algorithm was applied [40]. The LOH data were merged with CNAs in order to obtain the cnLOH, as previously reported [40].

The Partek workflow and the Hg19 (reference file) were used for the gene annotation (RefSeq). The observed CNAs were checked against the database of genomic variants (dBVar). Gene ontology (GO) enrichment and gene family analysis of gene sets was performed using the gene set enrichment analysis (GSEA) annotation tool (www.broadinstitute.org/gsea). Relevance of altered genes to currently known canonical pathways was investigated using the MetaCore Analytical suite (Pathways Maps, GeneGo Inc., Thomson Reuters, St Joseph, MI, USA, thomsonreuters.com/products_services/science/science_products/a-z/metacore). In particular, MetaCore was used to calculate the statistical significance (*P* value) of the probability of assembly from a random set of nodes (genes) of the same size as the input list (single experiment analysis). A *P<0.02* indicates a statistically significant non random association. The same software was used to characterize the top scored gene networks.

### Correlation to clinical data

The comparison of clinical parameters, such as age at diagnosis, tumor size, hormonal secretion pattern, tumor stage (ENSAT), Weiss score, and the proliferation index MIB1 (Ki67 index), and presence of distant metastasis between groups with or without genetic alterations were performed by appropriate statistical methods. The Fisher’s exact test or the Chi-square test was used to investigate the relationship between dichotomic variables, while a one-way ANOVA model or a two-sided *t* test was used to test continuous variables. Correlations between different parameters were evaluated by linear regression analysis. Survival analysis was calculated using the Kaplan-Meier method and differences between groups were assessed with log-rank statistics. Overall survival (OS) was defined as the time from the date of the primary tumor excision to date of death or last analysis. A multivariate regression analysis was performed by Cox proportional hazard regression model. Statistical analyses were performed using GraphPad Prism (version 4.0, La Jolla, CA, USA) and SPSS Software (PASW Version 19.0, SPSS Inc., Chicago, IL, USA). P values *<0.05* were considered as statistically significant.

### FISH analysis

Selected CNAs identified by SNP array results were validated on paraffin-embedded tumor tissue samples (12 ACA and 10 ACC) using fluorescence in situ hybridization (FISH) analysis according to previously published methods [45]. Specifically, BAC clones (Children’s Hospital Oakland Research Institute) were used. They were labeled with fluorophores, fragmented and hybridized together with the corresponding centromer-specific reference probe (Vysis, Abbott Molecular, IL, USA). The probes chosen for the region 5q-5p, largely amplified in ACC according to the SNP array results, were CSF1R/D5S23 and D5S721. A total of 100 interphase nuclei were analyzed with a Zeiss Axioskop 2 Microscope (Carl Zeiss MicroImaging) by two independent operators and a ratio of red signals (specific probes 5q-5p) to green signals (centromeric probes) was calculated. The cut-off for defining gains or losses was >40% cells.

### Quantitative real time PCR (qRT-PCR)

RNA was isolated from frozen tumor tissue samples for expression analysis (16 normal adrenal, 18 ACA, and 14 ACC) using the RNeasy Lipid Tissue Minikit (Qiagen) and reverse transcribed using the QuantiTect Reverse Transcription Kit (Qiagen). Predesigned Taqman® gene expression assays for *IGF2* gene (Hs00171254_m1) were purchased from Applied Biosystems (Darmstadt, Germany). Endogenously expressed *β-actin* (Hs9999903_m1) was used for normalization. 40 ng cDNA was used for each PCR reaction and each sample was performed in duplicate. Transcript levels were determined by using the TaqMan Gene Expression Master Mix (Applied Biosystems), the C1000 Thermal Cycler (CFX96 real-time system, Biorad) and the Bio-Rad CFX Manager 2.0 software. Cycling conditions were 95° for three min followed by 50 cycles of 95° for 30 sec, 60° for 30 sec, and 72° for 30 sec. Using the ∆CT method [46], the gene expression levels were normalized to those of the endogenous control β-actin, which was detected in all samples.

## Results

### Clinical data

The final study cohort included 46 adrenocortical tumor tissues with matched normal samples (42 from blood samples and 4 from adrenal tissue surrounding the tumors). Specifically, 24 patients with ACA (13F & 11M, mean age at diagnosis: 50±14 yrs, 16 cortisol-secreting) and 22 patients with ACC (14F & 8M, mean age at diagnosis: 46±14 yrs, 15 cortisol-secreting) have been investigated. The detailed clinical, pathological and corresponding genetic characteristics of the ACA and ACC groups are provided in Table ***1*** *.*

**Table 1:** Summary of clinical features and genetic alterations observed by the SNP microarray analysis of 46 patients with adrenocortical tumors.

	*Adenoma*	*Carcinoma*	*P*
N	24	22	
**Clinical parameters**			
Sex (M/F)	11/13	8/14	NS
Age (yrs) – median (range)	49.5 (17-71)	46.5 (18-72)	NS
Tumor size (cm) – median (range)	4.0 (2-11)	10.5 (4-23)	***<0.001***
Hormonal pattern			NS
Nonsecreting (n)	8	3	-
Cortisol-secreting (n)	16	15	-
Aldosteron-secreting (n)	-	1	-
Androgen-secreting (n)	-	3	-
Tumor stage (ENSAT)			-
ENSAT 1-2 (n)	-	12	-
ENSAT 3 (n)		4	-
ENSAT 4 (n)		6	-
**Genetic alteration**			
CNA - median (range)	43.5 (1-688)	132 (28-773)	NS
Gains - median (range)	27.5 (0-665)	68.5 (12-376)	NS
Losses - median (range)	5.5 (0-578)	72.5 (16-726)	***0.06***
Large CNA* – median (range)	7.0 (0-299)	62.5 (8-499)	***<0.01***
cnLOH - median (range)	0 (0-13)	4333 (0-8655)	***<0.0005***

CNA=copy number alterations, cnLOH= copy neutral loss of heterozygosity. * more than 100 Kb.

### SNP array profiling may differentiate benign from malignant adrenocortical tumors

#### Principal component analysis (PCA) and genomic clustering

**Figure 1:**
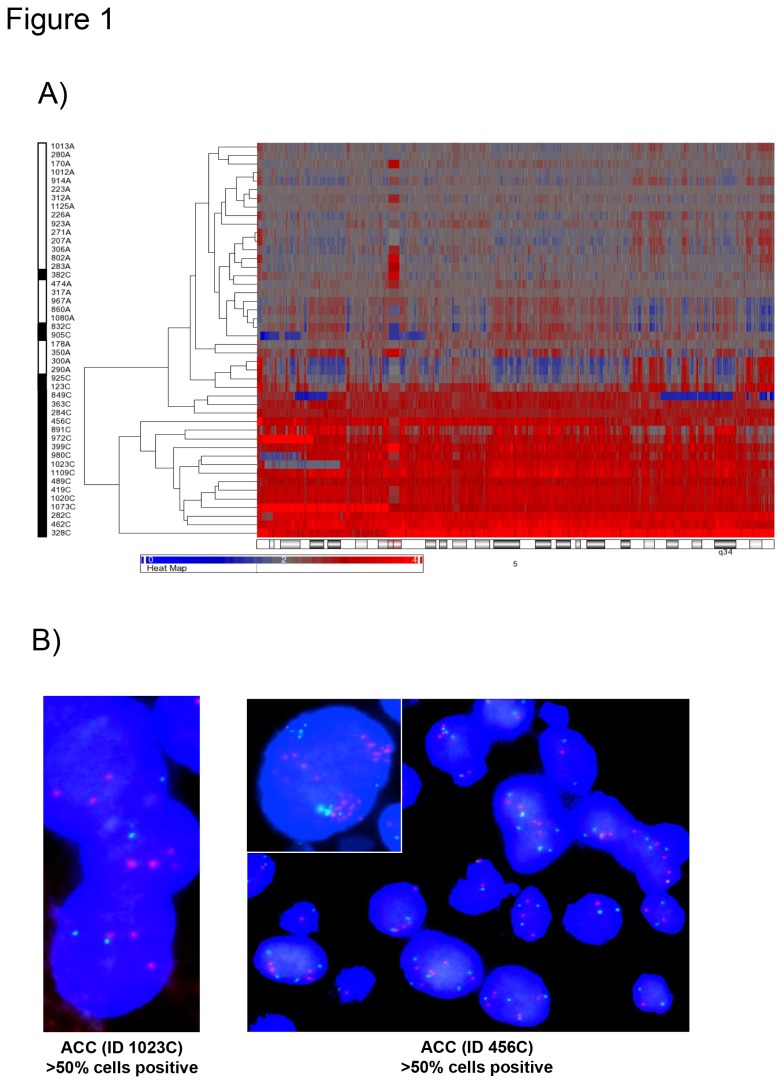
Copy number amplifications in chromosome 5 are specific for malignant adrenocortical tumors. A) Unsupervised euclidean distance genomic clustering (complete linkage) in 46 adrenocortical tumor samples (defined as sample ID). Best separation between adenoma (ACA, n=24, white) and carcinoma (ACC, n=22, black) obtained analyzing only the chromosome 5. B) Validation with FISH analysis of the copy number alterations detected with SNP array in chromosome 5. Two representative adrenocortical carcinomas (ACC, samples ID 456C and 1023C) with gains/amplifications at SNP array analysis validated by FISH (probe FISH_5q/5p-Probe: CSF1R/D5S23, D5S721). Gain of the red signal (specific probe) with respect to the green signal (centromer-specific reference probe) in more than 50% of cells. In black the ACC patients affected by ACC and in white the patients affected by ACA.

The PCA showed a good separation of the ACCs from the ACAs, previously classified according to established morphological criteria (Figure ***S1***). The unsupervised genomic clustering including the most altered chromosomes (i.e. chr 1, 5, 7, and 12) could separate well ACAs from ACCs. This was even more evident when considering only chr 5. In particular, 17/22 ACCs (77%) presented gains involving more than 60% of chr 5, while the ACAs had only few sparse small alterations (consisting of both gains and losses) (Figure ***1A***). Specifically, large amplifications at chr 5 at the SNP arrays analysis allowed us to recognize ACC with a sensitivity of 77.3% and a specificity of 100% (positive and negative predictive values: 100% and 82.2%, respectively).

The genetic alterations at the chr 5 were further validated by FISH analysis (Figure ***1B***), which confirmed the presence of 5p/5q amplifications in 10/10 ACCs (>50% of evaluated cells in 8/10 ACCs). In addition, 5p/5q amplifications were also observed by FISH in 3/12 ACAs (*P<0.05 vs* ACC), but only one benign tumor showed this alteration in >50% of evaluated cells. This adenoma also presented some histo-pathological signs of malignancy, such as large and highly pleomorphic nuclei, eosinophilic and pigmented cytoplasm, a central mylolipomatose metaplasia and a proliferation index (Ki67) of 2%. These results imply a role for the amplification of almost the entire chr 5 in adrenocortical tumorigenesis and suggest it as a new potential diagnostic marker.

No differences were observed in terms of clustering between cortisol-secreting and non cortisol-secreting tumors or among different tumor stages in ACC.

#### Paired CNA analysis

**Figure 2:**
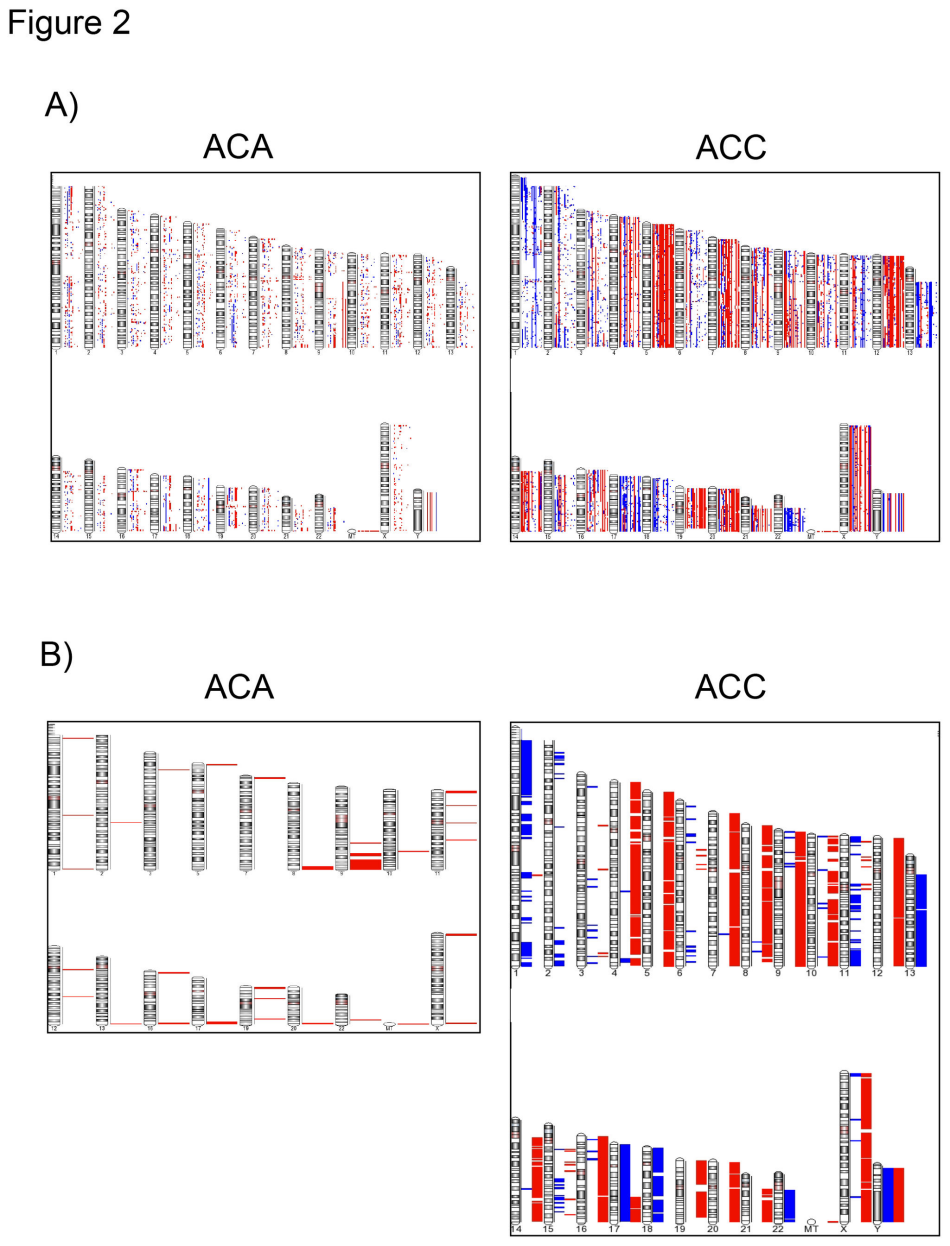
Whole-genome copy number alterations (CNA) in adrenocortical tumors. A) Genome-wide distribution of CNA in each adrenocortical tumor according to the genomic segmentation algorithm (Partek GS). ACA=adenoma (n=24, total number of CNA=3603). ACC=carcinoma (n=22, total number of CNA=5017). B) Genome-wide distribution of recurrent CNA in adrenocortical tumors. Minimal overlapping regions in at least 4 samples (MORs, frequency >15%). ACA=adenoma (n=24, total number of CNA=283). ACC=carcinoma (n=22, total number of CNA=3993). CN gains are represented in red and CN losses in blue.

All tumors had at least one aberration and the total number of CNAs was slightly higher in ACCs than in ACAs (median per sample: 132 *vs* 43.5, *P=0.19*, Table ***1***). However, 11/24 ACAs and 21/22 ACCs presented at least 50 CNAs along the entire genome (*P<0.05*). In comparison with benign tumors, the malignant ones showed a higher proportion of CN losses (55% *vs* 31%, *P=0.001*) and of large CNAs (>100 Kb, 56% vs 25%, *P<0.01*). The genome-wide distribution of the CNA in each tumor sample is provided in the Figure ***2A***. The entire list of detected CNAs and their characteristics, including corresponding genes is provided in Table ***S1***. No significant correlation was observed between the total number of CNA and the clinical parameters.

Aiming to focus on genetic alterations with possible biological significance, we further considered only the CNAs found in chromosomal regions coding for known genes and were observed in at least 4 samples (minimal overlapping regions, frequency >15%, Figure ***2B***, Table ***S2***). These recurrent alterations were more frequent in ACC (1680 gains and 951 losses) than in ACA (94 gains and no losses). Only few recurrent CN gains, involving 13 chromosomal regions, were detected only in benign tumors, but not in ACC. Most of them were microalterations, except those at 1p36.33, 8q24.3 (including *CYP11B2*), and 11p15.5 (including *IGF2* and *INS*), which ranged from 1000 to 4000 Kb. Interestingly, most of these regions were also altered among ACCs, but they were affected by losses instead of gains (excluding chr 2q24.2, 3p24.1, 8q24.3, 10q24.32).

#### LOH analysis

**Figure 3:**
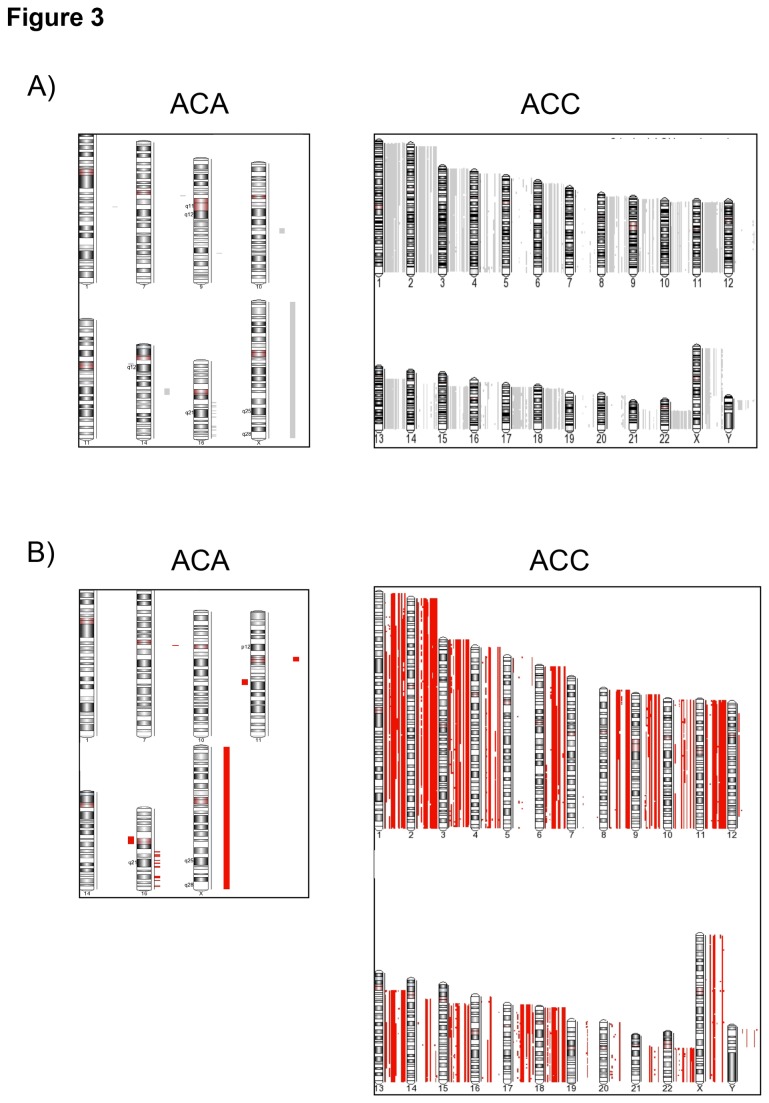
Whole-genome loss of heterozygosity (LOH) events in adrenocortical tumors. A) Genome-wide distribution of LOH events in each adrenocortical tumor according to the hidden markov model (Partek GS). ACA=adenoma (n=24, total number of LOH=23). ACC=carcinoma (n=22, total number of LOH=2439). B) Genome-wide distribution of copy neutral LOH identified by merged analysis between copy number alteration and LOH analysis.

**Figure 4:**
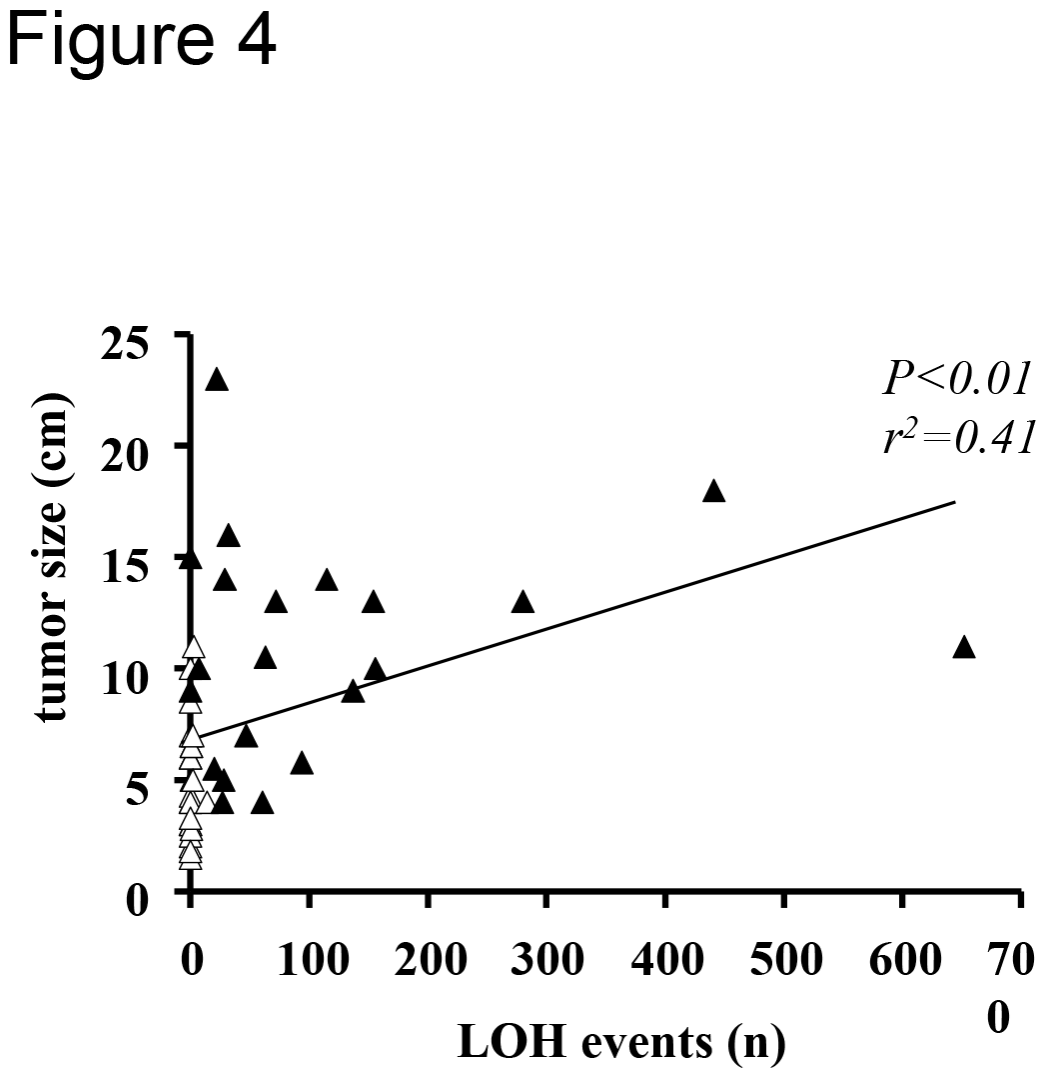
Correlation between the total number of LOH events and tumor size. All the evaluated adrenocortical tumors (n=46) were included in the analysis by linear regression analysis. White triangles = adenomas (n=24), black triangles = carcinomas (n=22).

**Figure 5:**
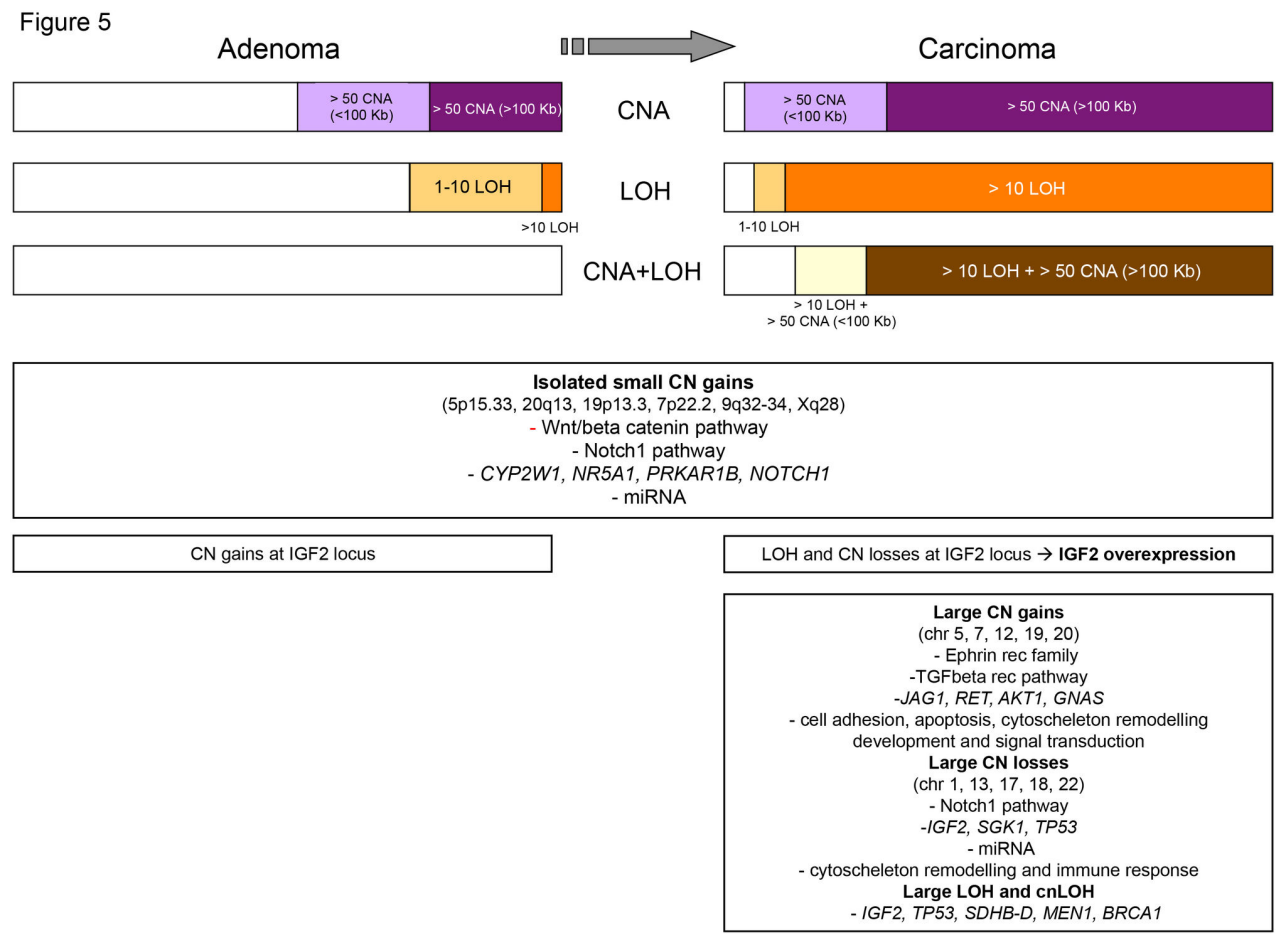
Schematic representation of the genetic alterations detected by SNP array analysis in adrenocortical tumors. In the upper panel, the percentage of samples affected by different alterations is reported. Interestingly, the simultaneous presence of numerous LOH and large CNA was observed only in malignant tumors (n=22). In the lower panel, some relevant specific alterations in candidate genes and pathway are reported. In particular, some alterations were observed in all tumors (i.e. isolated small CN gains), while others were found only in carcinomas. CNA=copy number alterations, LOH=loss of heterozygosity) detected by SNP array analysis in adrenocortical adenomas (n=24) and carcinomas (n=22).

23 LOH events were detected in 7/24 ACA (29%), none being present in more than one sample, while a high number of LOH events were distributed over the whole genome in 20/22 ACCs (91%, Table ***1*** and Figure ***3A***). The total number of LOH significantly correlated with tumor size considering all tumors together (*P<0.01*, r=0.41, Figure ***4***). Merging the CNA with the LOH analysis, we observed that in ACAs only one LOH event was associated with a CN loss (at Xq21.1, noncoding), while 5 were associated with a CN gain and 17 were copy neutral LOH (cnLOH) events (Figure ***3B***). A similar proportion of cnLOH was found among LOH also for ACCs (n=5848, Figure ***3B***). The simultaneous presence of more than 50 large CNA (>100 Kb) and more than 10 cnLOH events was specific for ACC (18/22 ACC vs 0/24 ACA, sensitivity 82% and specificity 100%; positive and negative predictive values: 100% and 85.7%, respectively, Figure ***5***).

### Specific gains/microamplifications might be early events in tumorigenesis

**Figure 6:**
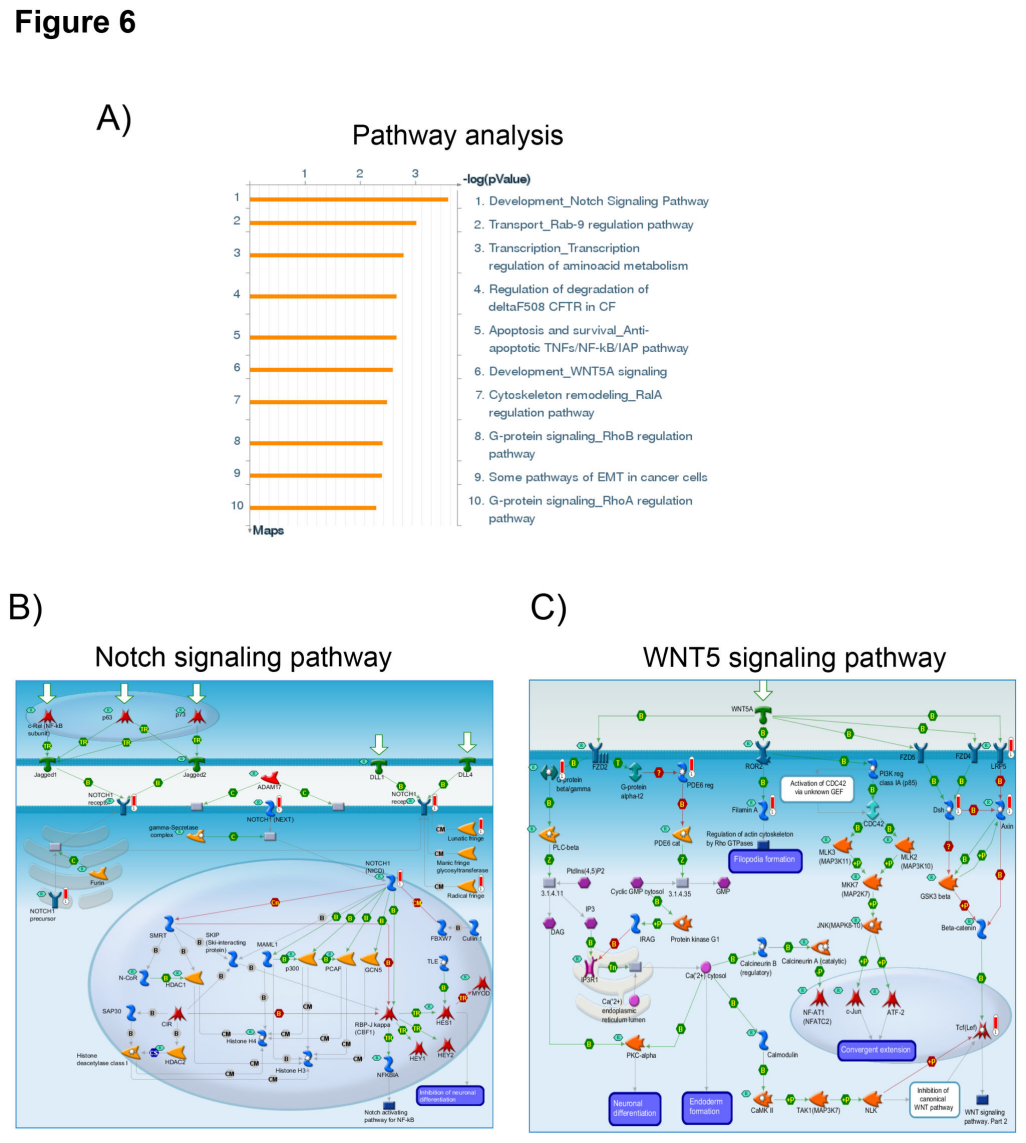
Pathway analysis for genes frequently affected by copy number alterations in both adenomas and carcinomas. A) List of the most significantly altered pathways according to the GeneGo analysis (Meta Core Analytical suite) including all the genes (n=696 annotated genes) with copy number alterations observed in at least 4 samples (recurrent CNA) in both adenomas and carcinomas. The P values are expresses as logarithmic scale. B) Graphical representation of the Notch signaling pathway map (gene ontology enrichment, *P=2.913* ^*e-4*^). C) Graphical representation of the WNT5 signaling pathway (gene ontology enrichment, *P=3.256* ^*e-3*^). Genes with gains are underlined in red and genes with losses in blue. Detailed legend is available at http://pathwaymaps.com/pdf/MC_legend.pdf.

More than 70% of recurrent CN gains detected in the ACA were present also in ACC (Table ***S3***). Specifically, we observed 81 common CN gains, mostly involving chr 5p13.33, 9q32-34, 16p13.3, and 19p13-12, among whom 43 were microamplifications. These shared altered regions code for 696 genes recognized in GO, the most interesting being reported in Table ***2***. Among these genes, 11 cytokines and growth factors, 41 transcription factors (including *NR5A1* coding for steroidogenic factor 1), 16 protein kinases, 11 known oncogenes (including *HRAS* and *NOTCH1*) and 4 known tumor suppressor genes were recognized. The Notch1 signaling was recognized as the most frequently altered pathway (*P<0.005*), followed by the anti-apoptotic TNFs/NF-kB/IAP pathway (*P<0.05*) and by the WNT5A signaling (*P<0.05*) (Figure ***6A-C***), while the gene network analysis identified the Wnt receptor signaling as the most relevant network (Figure ***2S*** ***AandB***).

**Table 2:** List of chromosomal regions affected by most frequent copy number gains in both adrenocortical adenomas (ACA, n=24) and carcinomas (ACC, n=22). A selection of the most interesting corresponding genes is also reported*.

*Cytoband*	*Naltered* * ACA*	*Naltered* * ACC*	*Selected corresponding genes*
5p15.33	5	18	AHRR, PDCD6, SDHA, NKD2
	5	16	TERT, CLPTM1L
	5	17	LPCAT1
20q13.3-	6	15	BIRC7, EEF1A2, MIR1-1, NPBWR2, NTSR1, PTK6, TNFRSF6B, TPD52L2
19p13.3	4	11	CIRBP, STK11
	4	10	GNG7
	4	10	ABCA7, BSG, **FSTL3**, KISS1R, APC2, DOT1L, GADD45B, MKNK2, **TCF3**
7p22.2	5	11	GNA12
	6	11	NUTD1
7p22.2-22.3	7	11	CYP2W1, GPER, GPR146, PDGFA, PRKAR1B
9q32	4	11	PRPF4, SLC31A1
9q32-33.1	4	11	ORM1, TNFSF15
9q33.1	4	10	DEC1
9q33.2	4	11	DAB2IP
	4	10	PTGS1
9q33.3	4	10	HSPA5, SCAI
9q33.32-33.33	4	10	NEK6, **NR5A1**
9q33.3-34.11	4	10	CDK9, ENG, LCN2
	4	8	SPTAN1
	4	9	**ABL1**, ASS1, ENDOG, **FNBP1**, **NUP214**, PKN3, **SET**
9q34.13-34.2	4	10	RALGDS, RPL7A, TSC1
9q34.2	5	10	**BRD3**, WDR5
	4	10	VAV2
9q34.2-34.3	5	10	RXRA
9q34.3	5	9	EGFL7, **NOTCH1**, TRAF2, NRARP
9q22.31	4	10	WNK2
Xq28	5	9	CTAG1B, FLNA, G6PD, L1CAM, TAZ, TKTL1
16q24.3	5	9	ANKRD11
	6	8	FANCA, GAS8, TUBB3
	5	8	CDK10, CHMP1A, DPEP1
Xp22.33	6	6	**CRLF2**, P2RY8
16p13.3	7	6	SSTR5
	7	5	AXIN1
	6	5	E4F1
17q25.3	4	4	ASPSCR1

* Genes known to be involved in tumorigenesis, response to cancer treatment or steroidogenesis.

Bold=known oncogenes.

In addition, several of these genes commonly gained in ACA and ACC are known or supposed to be associated with the Wnt/beta catenin signaling pathway (i.e. *APC2, CSNK1G2, AXIN1, NKD2, CSNK1P, EMD, VAV2*), the Notch signaling pathway (i.e. *LFNG2, RFNG, NRARP, NKD2, MIR199B, NDUFS7, TCFLS*), the mTOR pathway (i.e. *MAPKAP1, TSC1, TSC2, RPTOR, PPAPDC3, MLST8*) or the p53 pathway (i.e. *E4F1, GPS1, MRPL41, ARID3, GAMT*). A number of these genes (n=18) have been previously associated with resistance to chemotherapy in other cancer types (i.e. among others *PDCD6, ENDOG, BIRC7, CIRBP, ABCA7, MBD3, TCF3, ORM1, FLNA, NEX6, TUBB3, ABCA2*). Finally, 26 microRNAs were also on the list of common altered gene as were some other interesting genes, such as the orphan enzyme *CYP2W1* and the *PRKAR1B* coding for the regulatory subunit 1 beta of the protein kinase A.

Finally, 121 cnLOH events, involving 61 different regions on chr 1p, 1q, 9, 10q, 11p, 14q, 16q, Xp, and Xq, were similar between ACA and ACC (see Table ***S4***). They code for 618 genes recognized in GO, including 10 cytokines and growth factors, 59 transcription factors, 16 protein kinase, 8 oncogenes (*FOXO4, MSN, MYST4, SSX1, SSX2, SSX4, STIL, TFE3*), 3 tumor suppressor genes (*FAM123B, KDM5C*, *KDM6A*), and 64 microRNAs (including MIR503). Genes commonly affected by cnLOH were mainly involved in neurotrophin family signaling, pathways related to cell cycle (sister chromatid cohesion), immune response (IL2 and IL3 activation), and cell adhesion (ECM remodelling). The gene network analysis identified the toll-like 1 and 2 signaling pathway as the most frequently affected (*P<0.0005*).

### Genetic alterations at the 11p15.5 locus as example of adenoma-carcinoma sequence

As expected according to the current knowledge, a large percentage of our ACCs presented an LOH event in a segment at the imprinted chr 11p15.5 locus, which included the genes *IGF2, IGF2AS, INS, INS-IGF2*, and *MIR483*. In particular, 12/22 cases had a cnLOH (54% of total) and other 5 cases had an LOH associated with CN loss (23%). Interestingly, we also observed in the same locus an isolated CN gain in 2/22 ACC (9%) and in 6/24 ACA (25%).

**Figure 7:**
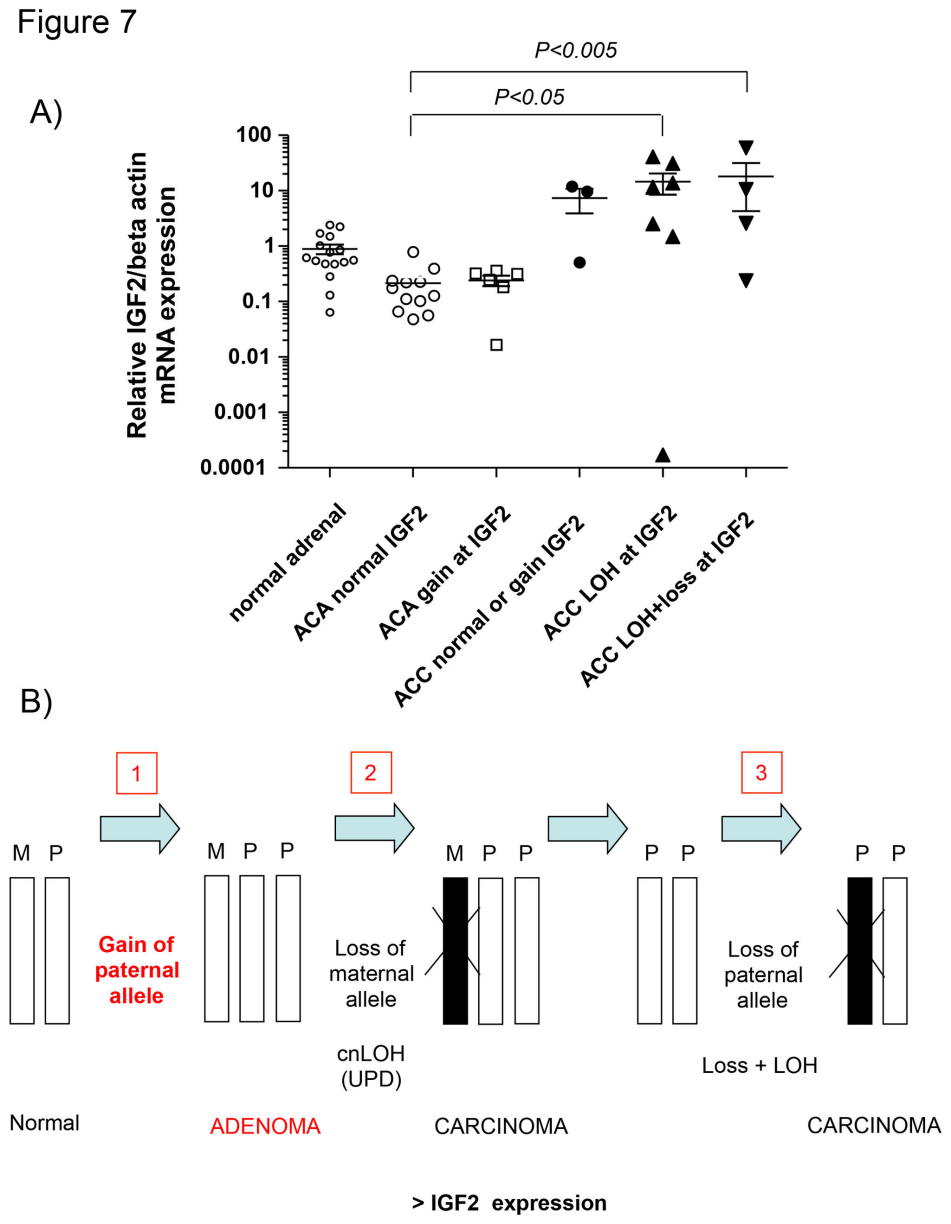
Relationship between genomic alterations at chr 11p15.5 and IGF2 gene expression in adrenocortical tumors. A) Relative mRNA expression levels evaluated by qRT-PCR for *IGF2* gene according to the copy number status observed with SNP array analysis in normal adrenals (n=16), adenomas (n=18) and carcinomas (n=14). The gene actin beta was used as a loading control (reference gene). Data expressed as logarithmic scale. *P=0.0003* by one-way ANOVA. B) Schematic representation of the genetic alterations at the 11p15.5 imprinted locus and their impact on IGF2 expression levels. In particular, we firstly report frequent copy number gains in adenomas, leading to a normal IGF2 expression, and we confirm frequent copy neutral LOH events in carcinoma, leading to an increased IGF2 expression, suggesting a progressive multi-step genetic derangement in this locus. We also demonstrate a further allele loss in carcinoma (LOH + CN loss) without effects on IGF2 expression.

The relative IGF2 mRNA expression was also evaluated in our samples and correlated with the CN status. Specifically, we found that the IGF2 levels were similar between ACAs and normal adrenals, independently from the genetic alterations. On the other hand, IGF2 expression was significantly increased in ACC (*P<0.001*), especially in those samples affected by cnLOH or LOH associated with CN losses (Figure ***7A***), thus suggesting a possible multistep genomic aberration at this locus (i.e. first a duplication of the paternal allele followed by the loss of the maternal allele and as late step by the loss also of one paternal allele). However, probably due to the small number of patients included in each subgroup, we were not able to find any significant correlation between the specific genetic alterations at the IGF2 locus and the clinical data (such as tumor size or stage).

### Enrichment analysis shows specific altered pathways in ACC

The gene family analysis performed for all the frequently altered genes is reported separately for adenomas and carcinomas in the Table ***S5***.

We focused on the aberrations specific for ACC and included only the genes specifically altered in ACC in further analysis. Among the gained genes (n=11415 recognized in GO), we identified over 160 known oncogenes (including *ABL1, AKT1-2, BRAF, EGFR, FGFR1-3, GNAS, JAK2-3, KIT, MYC, NOTCH1*, and *RET*). On the other side, 20 tumor suppressor genes (including *BRCA1-2, ERCC5, MEN1, RB1* and *TP53*) were identified among the lost genes (n=3717 recognized in GO) and 67 tumor suppressor genes (including *ERCC3*, and *TP53*) were identified among the genes affected by cnLOH.

The most frequently altered pathways were related to cell adhesion, apoptosis and survival, cytoskeleton remodeling, development, and signal transduction when considering the gained genes (Figure ***S3 A***) and mainly involved cytoskelton remodeling and immune response when considering the lost genes (Figure ***S3 B***). Finally, the pathway analysis showed as most affected by cnLOH the cytoskeleton remodeling (TGF, WNT, and cytoskeleton remodeling, *P<0.0001*), immune response (HSP60 and 70/TLR, *P<0.0001*, and HMGB1/TLR signaling, *P<0.001*), and cell adhesion (chemokines and adhesion, *P<0.001*).

### A specific CNA pattern might have prognostic value in ACC

**Figure 8:**
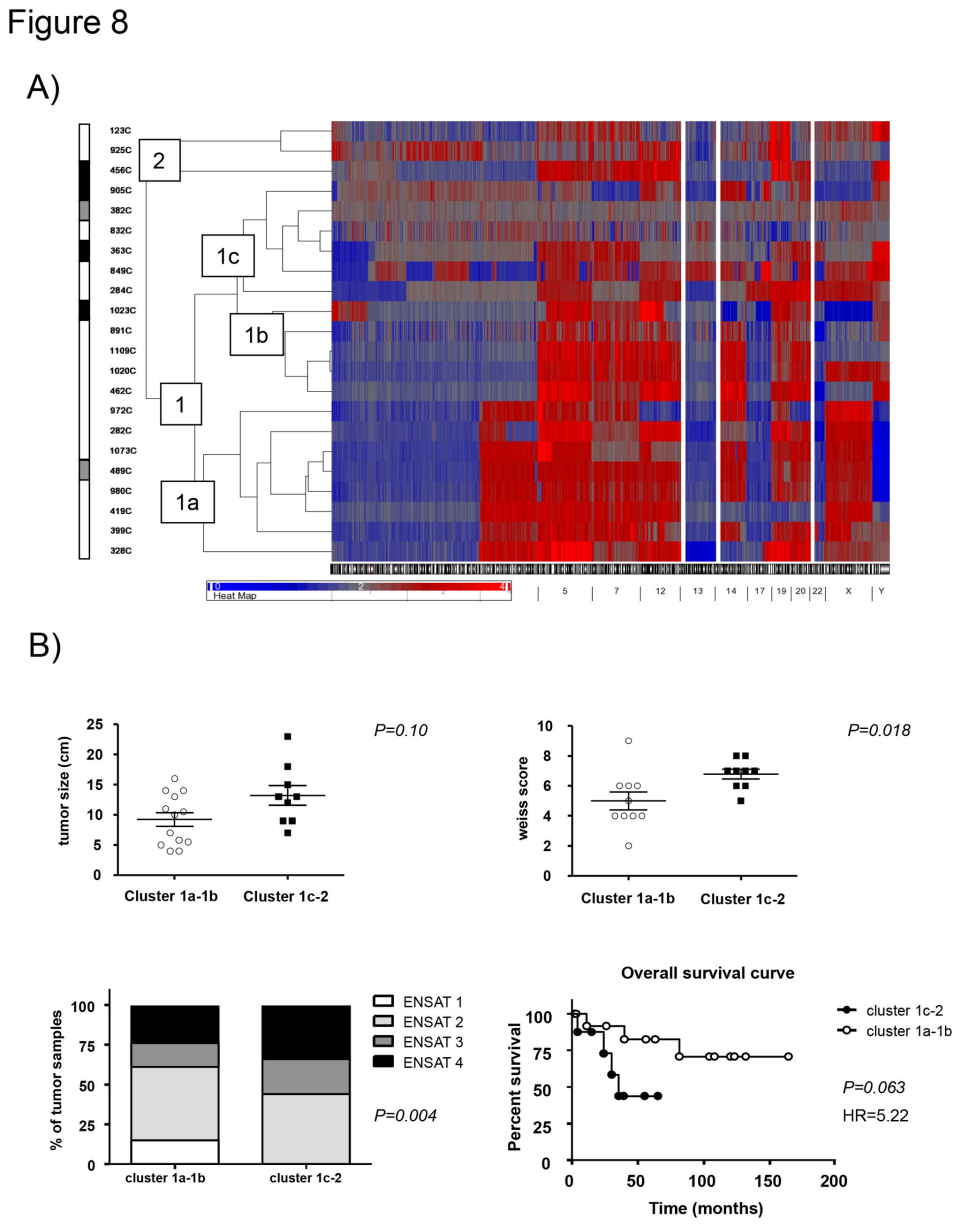
Relationship between genomic alterations and clinical outcome in malignant adrenocortical tumors. A) Unsupervised euclidean distance genomic clustering (complete linkage) in 22 carcinomas. Best cluster obtained including a specific combination of chromosomes (i.e. chr 1, 2, 4, 5, 7, 12, 13, 14, 17, 18, 20, 22, and X). B) Relationship between the genetic clusters and the clinical parameters: tumor size and Weiss score (Mann-Withney test), ENSAT tumor stage (Fisher’s test) and overall survival (Kaplan-Meyer curve and log-rank test). In black the patients with a worst prognosis (overall survival < 24 months), in grey the patients with an intermediate prognosis (overall survival 24-48 months), and in white the patients with a good prognosis (overall survival > 48 months or still alive at the last follow up).

Searching for a possible prognostic value of genetic alterations, we also performed an unsupervised genomic clustering including only the ACCs. We identified a specific combination of chromosomal alterations which allowed us to separate the malignant tumors into different clusters (see Figure ***8A***). In particular, the tumors included in the clusters 1a (n=8) and 1b (n=5) behaved quite similar presenting several large amplification at chr 5, 7, 12, and 19, and large deletions at chr 1, 2, 13, 17, and 22, while the 9 other tumors showed an extremely variable pattern of genetic alterations. The relationship with the clinical data showed that the first group (clusters 1a+1b) had smaller tumor size (9.2±4.2 vs 13.2±5.0 cm, *P=0.10*), lower Weiss score (5.0±1.9 vs 6.8±1.0, *P=0.018*) and lower ENSAT tumor stage (ENSAT 1-2: 61% vs 44%, *P=0.016*). In addition, they also had a longer overall survival in comparison with the other cluster groups (median survival: 72.2 vs 35.4 months, *P=0.063*, HR=5.22, 95%CI=0.91-25.9, Figure ***8B***). However, this difference was lost when including the genetic pattern in a multivariate analysis together with the ENSAT tumor stage and the Ki67 index.

## Discussion

We here provide for the first time a genome-wide high-resolution overview of chromosomal changes in a large series of adrenocortical tumors, including adenomas and carcinomas. In a previous pilot study we already observed in a small group of benign cortisol-secreting tumors frequent CN gains in newly reported chromosomal regions (i.e. 8q24.3, 11p15.5, Xq28) and promising candidate genes or pathways potentially involved in early tumorigenesis [40]. In the present paper, we identify some pathways, such as Wnt/β-catenin and Notch signaling, commonly altered in ACA and ACC, strengthening our previous hypothesis that these pathways are involved in early tumor pathogenesis. Moreover, we recognize some genetic aberrations specific for ACC, such as the amplification of the chr 5, recurrent large CNA (mostly losses), and frequent LOH and cnLOH events distributed all over the genome, that could be used as markers of malignancy. In addition, we identify a specific genetic pattern associated with a better prognosis.

### Alterations suggestive for an adenoma-carcinoma sequence (similar between ACA and ACC)

A recent study by Heaton et al. inducing stabilized beta-catenin and elevated Igf2 expression in mice suggested a multistep model whereby normal adrenal glands become first hyperplastic and then progress to ACA and ACC [10]. Single cases with the presence of an aggressive ACC embedded within ACA tissue are also suggestive for such a multistep progression [7,47]. However, due to the obvious discordance between the frequency of ACA and the rarity of ACC most clinical researcher believe that there is not such a sequence like it is well established in colon tumors [48]. Several of our present findings support now the evidence of an adenoma carcinoma sequence for adrenocortical tumors.

First, about half of our ACA samples and almost all the ACC samples presented in average similar numbers of CNA along the entire genome. Among the benign tumors, small isolated CN gains were the most frequent genetic alterations and almost all of them were also present in several ACC (Table 2). Many of these aberrations have already reported in other cancer types. For instance, frequent gains at 5p13.3 and 20q11-13 have been described in early stage NSCL cancer and in colorectal tumors [49,50,51], suggesting a potential role of these as a first step in tumor progression. In addition, gains at chr 7p22, 9q22.3, and Xq28 have been already described in other early-stage cancers [50,52,53,54,55]. Finally, recurrent gains at 8q24, 9q33-34 and 19p13-q13 have been also similarly reported in childhood adrenocortical tumors [41].

Second, some canonical pathways, such as the Wnt/beta catenin and the Notch1 signalling, were frequently affected by CN gains in both ACA and ACC. The Wnt/beta catenin pathway is known to be activated in adrenocortical tumors and to be involved in adrenal development and tumorigenesis [56], but until now no CNA have been reported in this pathway. On the other hand, the Notch signalling pathway has not been yet directly associated with tumor development and progression in ACC. Furthermore, Notch and Wnt/beta-catenin signalling often intersect in stem and progenitor cells and regulate each other transcriptionally, the biological outcome of signalling through each pathway often depending on the context and timing. Thus, these findings need to be investigated more in details.

Finally, we obtained interesting results about the imprinted locus at chr 11p15.5. Specifically, we observed in this region (including the genes *IGF2, IGF2AS, INS, INS-IGF2*, and *MIR483*) frequent cnLOH or LOH associated with CN loss in ACC (54% and 23% of cases, respectively) and CN gains in 25% of ACA. Thus, we could hypothesize that a CN gain at this locus could be an early alteration in a multi-step tumor progression (already present in benign tumors), while the following loss of an allele leading to a cnLOH could represent a second hit, leading to the increased IGF2 expression only in malignant tumors. An additional CN loss could then here somehow represent a final step, but without further modifying the IGF2 expression levels (Figure ***7B***). These findings are of particular interest because the human chr 11p15.5 contains a cluster of imprinted genes (organized in two neighbouring imprinted domains, the *IGF2/H19* and the *KCNQ1OT1/CDKN1C*) that play a crucial role in the development of many organ systems, including the adrenal cortex. Aberrations of the 11p15 region were found in both Beckwith–Wiedemann and Silver–Russell syndromes [57], and were also reported by SNP array profiling in childhood adrenocortical tumors [41]. Loss of imprinting has also been reported in colorectal carcinomas, Wilm’s tumor, esophageal carcinoma, childhood acute lymphoblastic leukemia, and prostate cancer. In cells that express both parental IGF2 alleles, the increase in IGF2 production may be a mechanism for promoting cancer development [58]. Structural and functional abnormalities at 11p15 are associated with the malignant phenotype in sporadic adrenocortical tumors [59] and *IGF2* expression is dramatically up-regulated in at least 80% of sporadic ACCs, compared with either ACA or normal adrenal tissue [60,61]. Finally, a recent paper showed that a mouse model with a combination of stabilized β-catenin and elevated IGF2 expression presented larger adrenal glands, displayed earlier onset of hyperplasia, and developed more frequent macroscopic adenomas (as well as one carcinoma) than the models with only one alteration, suggesting the need of an accumulation of a second or multiple alterations for tumorigenesis [10]. Similarly, another study analysed the role of IGF2 in transgenic mouse models and demonstrated that IGF2 over-expression even in combination with costitutive β-catenin activation, is only a mild contributor to malignant adrenocortical tumorigenesis, suggesting the involvement of multiple other factors [62]. Thus, we postulate a multi-step adenoma carcinoma sequence for adrenocortical tumors, although we are aware that the progression of adenoma to carcinoma is a rare event.

### Alterations potentially useful as diagnostic markers (different between ACA and ACC)

A series of genetic alterations detected with the SNP array analysis were found to be unique in malignant tumors. For instance, the amplification of more than 60% of the chr 5 was observed only in ACC. This finding was also confirmed at the FISH analysis, which could be suggested as a cheap and fast method for the identification of chr 5 amplification in adrenocortical tumors. Similar findings (i.e. CN gain of chr 5p) have been already reported in other cancer types, such as the cervical cancer [63], and also in adrenocortical cancer, but only together with other chromosomes (i.e. 4, 7, 12 and 19 [41,64]) and not as playing a major role. Thus, importantly, this kind of alteration could be used as a new differential diagnostic marker (sensitivity 77.3% and specificity 100%).

As far as the specific CN analysis is concerned, recurrent CN losses were completely absent in ACAs, which shows that they are rather specific for ACCs. In addition, the simultaneous presence of more than 50 large CNA (>100 Kb) and more than 10 LOH events resulted to be highly specific for ACC (sensitivity 82% and specificity 100%, Figure ***5***). This genetic pattern might be also used as a new marker for malignancy.

In terms of enrichment analysis, over 160 gained oncogenes, 20 lost tumor suppressor genes and 67 tumor suppressor genes affected by cnLOH were identified only in carcinomas. Some of these genes (i.e. *MYC, MDM2, PDGFRA*, and *KIT* among gained genes and *RB1* and *TP53* in lost genes) have been found also in childhood tumors [41], suggesting some common genetic alterations between children and adults. The most frequently altered pathways specific for malignant tumors were related to cell adhesion, apoptosis and survival, cytoskeleton remodeling, development, signal transduction, and immune response.

### Alterations potentially useful as prognostic factors (related to survival in ACC)

A specific combination of chromosomes with large CNA (i.e. large amplification at chr 5, 7, 12, and 19, and large deletions at chr 1, 2, 13, 17, and 22) was associated with a better prognosis, while a random pattern of alterations in the whole-genome seem to be correlated with a poorest survival. These findings may suggest a relationship between a specific genetic pattern and the overall clinical outcome, but need to be validated in a larger patient cohort and better investigated with further experiments.

In conclusion, by using high-resolution SNP array analysis, we identified some genes and pathways, such as the Notch signaling, which were similarly altered in ACA and ACC. Our findings suggest a potential major role for these alterations in early tumorigenesis and provide new insight on a postulated adenoma-carcinoma sequence in adrenocortical tumors. In general, our study can serve as a database for the identification of interesting genes and pathways involved in adrenal tumor development and progress. Moreover, some genetic alterations, such as large CNA (including the amplification of the chr 5 and huge losses) and LOH events, were restricted to the ACC and could serve as diagnostic markers for malignancy.

## Supporting Information

Figure S1 **Principal component analysis for the SNP array analysis of 46 adrenocortical tumors (24 adenoma in blue and 22 carcinoma in red) by Partek Genomic Suite Software.**
(TIF)

Figure S2 **Enrichment analysis including the genes with copy number alterations observed only in carcinomas (recurrent CNA, in at least 4 samples).** A) Analysis generated including genes affected by copy number gains (n=11414). B) Analysis generated including genes affected by copy number gains (n=3717). Gene family analysis by GSEA; pathway and gene process analysis by GeneGo (Meta Core Analytical suite, P values expresses as logarithmic scale; detailed legend available at http://pathwaymaps.com/pdf/MC_legend.pdf.).
(PPT)

Figure S3 A) Results of the generation of biological networks using Analyze Networks (AN) algorithm with default settings. The gene content of the uploaded files (genes with LOH events observed in at least 4 samples in carcinomas, n=11415) is used as the input list. This is a variant of the shortest paths algorithm with main parameters of 1. relative enrichment with the uploaded data, and 2. relative saturation of networks with canonical pathways. In this workflow the networks are prioritized based on the number of fragments of canonical pathways on the network. B) Graphical representation of the top scored (by the number of pathways) analysed network (positive regulation of the macromolecule metabolic process). Thick cyan lines indicate the fragments of canonical pathways. Up-regulated genes are marked with red circles; down-regulated with blue circles. The 'checkerboard' color indicates mixed expression for the gene between files or between multiple tags for the same gene. Detailed legend is available at http://pathwaymaps.com/pdf/MC_legend.pdf.
(PPT)

Table S1 **List of all the copy number alterations detected in 46 adrenocortical tumors by SNP array profile, including the relative chromosomal regions, length, type of alteration, number of markers, intensity score (mean) and corresponding genes.** A) adenomas (n=24); B) carcinomas (n=22).
(XLS)

Table S2 **List of all the copy number alterations detected in at least 4 samples among 46 adrenocortical tumors by SNP array profile (minimal overlapping regions, frequency > 13%), including the relative chromosomal regions, length, type of alteration, intensity score (average) and corresponding genes.** A) adenomas (n=24); B) carcinomas (n=22).
(XLS)

Table S3 **List of copy number alteration (all gains) in common between adrenocortical adenomas (n=24) and carcinomas (n=22), including the chromosomal regions, length of intersection, overlap percentage and corresponding genes.**
(XLS)

Table S4 **List of loss of heterozygosity (LOH) events in common between adrenocortical adenomas (n=24) and carcinomas (n=22), including the chromosomal regions, overlap percentage and corresponding genes.**
(XLS)

Table S5 **Gene family analysis (GSEA) of all the most frequently altered genes observed at the SNP array analysis in adrenocortical tumors, subdivided into adenomas and carcinomas.**
(DOC)

## References

[1] Bilimoria KY, Shen WT, Elaraj D, Bentrem DJ, Winchester DJ et al. (2008) Adrenocortical carcinoma in the United States: treatment utilization and prognostic factors. Cancer 113: 3130-3136. doi:10.1002/cncr.23886. PubMed: 18973179. 18973179 10.1002/cncr.23886

[2] Allolio B, Fassnacht M (2006) Clinical review: Adrenocortical carcinoma: clinical update. J Clin Endocrinol Metab 91: 2027-2037. doi:10.1210/jc.2005-2639. PubMed: 16551738. 16551738 10.1210/jc.2005-2639

[3] Libè R, Fratticci A, Bertherat J (2007) Adrenocortical cancer: pathophysiology and clinical management. Endocr Relat Cancer 14: 13-28. doi:10.1677/erc.1.01130. PubMed: 17395972. 17395972 10.1677/erc.1.01130

[4] Fassnacht M, Libé R, Kroiss M, Allolio B (2011) Adrenocortical carcinoma: a clinician’s update. Nat Rev Endocrinol 7: 323-335. doi:10.1038/nrendo.2010.235. PubMed: 21386792. 21386792 10.1038/nrendo.2010.235

[5] Icard P, Goudet P, Charpenay C, Andreassian B, Carnaille B et al. (2001) Adrenocortical carcinomas: surgical trends and results of a 253-patient series from the French Association of Endocrine Surgeons study group. World J Surg 25: 891-897. doi:10.1007/s00268-001-0047-y. PubMed: 11572030. 11572030 10.1007/s00268-001-0047-y

[6] Abiven G, Coste J, Groussin L, Anract P, Tissier F et al. (2006) Clinical and biological features in the prognosis of adrenocortical cancer: poor outcome of cortisol-secreting tumors in a series of 202 consecutive patients. J Clin Endocrinol Metab 91: 2650-2655. doi:10.1210/jc.2005-2730. PubMed: 16670169. 16670169 10.1210/jc.2005-2730

[7] Bernard MH, Sidhu S, Berger N, Peix JL, Marsh DJ et al. (2003) A case report in favor of a multistep adrenocortical tumorigenesis. J Clin Endocrinol Metab 88: 998-1001. doi:10.1210/jc.2002-021117. PubMed: 12629075. 12629075 10.1210/jc.2002-021117

[8] Barzon L, Sonino N, Fallo F, Palu G, Boscaro M (2003) Prevalence and natural history of adrenal incidentalomas. Eur J Endocrinol 149: 273-285. doi:10.1530/eje.0.1490273. PubMed: 14514341. 14514341 10.1530/eje.0.1490273

[9] Cawood TJ, Hunt PJ, O’Shea D, Cole D, Soule S (2009) Recommended evaluation of adrenal incidentalomas is costly, has high false-positive rates and confers a risk of fatal cancer that is similar to the risk of the adrenal lesion becoming malignant; time for a rethink? Eur J Endocrinol 161: 513-527. doi:10.1530/EJE-09-0234. PubMed: 19439510. 19439510 10.1530/EJE-09-0234

[10] Heaton JH, Wood MA, Kim AC, Lima LO, Barlaskar FM et al. (2012) Progression to adrenocortical tumorigenesis in mice and humans through insulin-like growth factor 2 and beta-catenin. Am J Pathol 181: 1017-1033. doi:10.1016/j.ajpath.2012.05.026. PubMed: 22800756. 22800756 10.1016/j.ajpath.2012.05.026 PMC3432433

[11] Weiss LM, Medeiros LJ, Vickery AL Jr. (1989) Pathologic features of prognostic significance in adrenocortical carcinoma. Am J Surg Pathol 13: 202-206. doi:10.1097/00000478-198903000-00004. PubMed: 2919718. 2919718 10.1097/00000478-198903000-00004

[12] Ragazzon B, Libé R, Gaujoux S, Assié G, Fratticci A et al. (2010) Transcriptome analysis reveals that p53 and {beta}-catenin alterations occur in a group of aggressive adrenocortical cancers. Cancer Res 70: 8276-8281. doi:10.1158/0008-5472.CAN-10-2014. PubMed: 20959480. 20959480 10.1158/0008-5472.CAN-10-2014

[13] Ragazzon B, Assié G, Bertherat J (2011) Transcriptome analysis of adrenocortical cancers: from molecular classification to the identification of new treatments. Endocr Relat Cancer 18: R15-R27. doi:10.1530/ERC-10-0290. PubMed: 21208995. 21208995 10.1530/ERC-10-0220

[14] Jain M, Rechache N, Kebebew E (2012) Molecular markers of adrenocortical tumors. J Surg Oncol, 106: 549–56. PubMed: 22504887. 22504887 10.1002/jso.23119 PMC6959534

[15] Lehmann T, Wrzesinski T (2012) The molecular basis of adrenocortical cancer. Cancer Genet 205: 131-137. doi:10.1016/j.cancergen.2012.02.009. PubMed: 22559973. 22559973 10.1016/j.cancergen.2012.02.009

[16] Gicquel C, Boulle N, Logie A, Bourcigaux N, Gaston V et al. (2001) [Involvement of the IGF system in the pathogenesis of adrenocortical tumors]. Ann Endocrinol (Paris) 62: 189-192. 11353893

[17] Soon PS, Libe R, Benn DE, Gill A, Shaw J et al. (2008) Loss of heterozygosity of 17p13, with possible involvement of ACADVL and ALOX15B, in the pathogenesis of adrenocortical tumors. Ann Surg 247: 157-164. doi:10.1097/SLA.0b013e318153ff55. PubMed: 18156936. 18156936 10.1097/SLA.0b013e318153ff55

[18] Tissier F, Cavard C, Groussin L, Perlemoine K, Fumey G et al. (2005) Mutations of beta-catenin in adrenocortical tumors: activation of the Wnt signaling pathway is a frequent event in both benign and malignant adrenocortical tumors. Cancer Res 65: 7622-7627. PubMed: 16140927. 16140927 10.1158/0008-5472.CAN-05-0593

[19] Kjellman M, Kallioniemi OP, Karhu R, Höög A, Farnebo LO et al. (1996) Genetic aberrations in adrenocortical tumors detected using comparative genomic hybridization correlate with tumor size and malignancy. Cancer Res 56: 4219-4223. PubMed: 8797595. 8797595

[20] Zhao J, Speel EJ, Muletta-Feurer S, Rütimann K, Saremaslani P et al. (1999) Analysis of genomic alterations in sporadic adrenocortical lesions. Gain of chromosome 17 is an early event in adrenocortical tumorigenesis. Am J Pathol 155: 1039-1045. doi:10.1016/S0002-9440(10)65205-4. PubMed: 10514385. 10514385 10.1016/S0002-9440(10)65205-4 PMC1867020

[21] Dohna M, Reincke M, Mincheva A, Allolio B, Solinas-Toldo S et al. (2000) Adrenocortical carcinoma is characterized by a high frequency of chromosomal gains and high-level amplifications. Genes Chromosomes Cancer 28: 145-152. doi:10.1002/(SICI)1098-2264(200006)28:2. PubMed: 10824999. 10824999

[22] Sidhu S, Marsh DJ, Theodosopoulos G, Philips J, Bambach CP et al. (2002) Comparative genomic hybridization analysis of adrenocortical tumors. J Clin Endocrinol Metab 87: 3467-3474. doi:10.1210/jc.87.7.3467. PubMed: 12107267. 12107267 10.1210/jcem.87.7.8697

[23] Zhao J, Roth J, Bode-Lesniewska B, Pfaltz M, Heitz PU et al. (2002) Combined comparative genomic hybridization and genomic microarray for detection of gene amplifications in pulmonary artery intimal sarcomas and adrenocortical tumors. Genes Chromosomes Cancer 34: 48-57. doi:10.1002/gcc.10035. PubMed: 11921282. 11921282 10.1002/gcc.10035

[24] Gruschwitz T, Breza J, Wunderlich H, Junker K (2010) Improvement of histopathological classification of adrenal gland tumors by genetic differentiation. World J Urol 28: 329-334. doi:10.1007/s00345-010-0541-7. PubMed: 20364258. 20364258 10.1007/s00345-010-0541-7

[25] Almeida MQ, Harran M, Bimpaki EI, Hsiao HP, Horvath A et al. (2011) Integrated genomic analysis of nodular tissue in macronodular adrenocortical hyperplasia: progression of tumorigenesis in a disorder associated with multiple benign lesions. J Clin Endocrinol Metab 96: E728-E738. doi:10.1210/jc.2010-2420. PubMed: 21252250. 21252250 10.1210/jc.2010-2420 PMC3070257

[26] Stephan EA, Chung TH, Grant CS, Kim S, Von Hoff DD et al. (2008) Adrenocortical carcinoma survival rates correlated to genomic copy number variants. Mol Cancer Ther 7: 425-431. doi:10.1158/1535-7163.MCT-07-0267. PubMed: 18281524. 18281524 10.1158/1535-7163.MCT-07-0267

[27] Barreau O, de Reynies A, Wilmot-Roussel H, Guillaud-Bataille M, Auzan C et al. (2011) Clinical and Pathophysiological Implications of Chromosomal Alterations in Adrenocortical Tumors: An Integrated Genomic Approach. J Clin Endocrinol Metab, 97: E301–11. PubMed: 22112813. 22112813 10.1210/jc.2011-1588

[28] Szabó PM, Tamási V, Molnár V, Andrásfalvy M, Tömböl Z et al. (2010) Meta-analysis of adrenocortical tumour genomics data: novel pathogenic pathways revealed. Oncogene 29: 3163-3172. doi:10.1038/onc.2010.80. PubMed: 20305693. 20305693 10.1038/onc.2010.80

[29] Zsippai A, Szabó DR, Szabó PM, Tömböl Z, Bendes MR et al. (2011) mRNA and microRNA expression patterns in adrenocortical cancer. Am J Cancer Res 1: 618-628. PubMed: 21994902. 21994902 PMC3189823

[30] Ronchi CL, Sbiera S, Kraus L, Wortmann S, Johanssen S et al. (2009) Expression of excision repair cross complementing group 1 and prognosis in adrenocortical carcinoma patients treated with platinum-based chemotherapy. Endocr Relat Cancer 16: 907-918. doi:10.1677/ERC-08-0224. PubMed: 19240185. 19240185 10.1677/ERC-08-0224

[31] Fenske W, Völker HU, Adam P, Hahner S, Johanssen S et al. (2009) Glucose transporter GLUT1 expression is an stage-independent predictor of clinical outcome in adrenocortical carcinoma. Endocr Relat Cancer 16: 919-928. doi:10.1677/ERC-08-0211. PubMed: 19465749. 19465749 10.1677/ERC-08-0211

[32] Volante M, Sperone P, Bollito E, Frangipane E, Rosas R et al. (2006) Matrix metalloproteinase type 2 expression in malignant adrenocortical tumors: Diagnostic and prognostic significance in a series of 50 adrenocortical carcinomas. Mod Pathol 19: 1563-1569. doi:10.1038/modpathol.3800683. PubMed: 16980949. 16980949 10.1038/modpathol.3800683

[33] Sbiera S, Schmull S, Assie G, Voelker HU, Kraus L et al. (2010) High diagnostic and prognostic value of steroidogenic factor-1 expression in adrenal tumors. J Clin Endocrinol Metab 95: E161-E171. doi:10.1210/jc.2010-0653. PubMed: 20660055. 20660055 10.1210/jc.2010-0653

[34] Doghman M, Karpova T, Rodrigues GA, Arhatte M, De Moura J et al. (2007) Increased steroidogenic factor-1 dosage triggers adrenocortical cell proliferation and cancer. Mol Endocrinol 21: 2968-2987. doi:10.1210/me.2007-0120. PubMed: 17761949. 17761949 10.1210/me.2007-0120

[35] LaFramboise T (2009) Single nucleotide polymorphism arrays: a decade of biological, computational and technological advances. Nucleic Acids Res 37: 4181-4193. doi:10.1093/nar/gkp552. PubMed: 19570852. 19570852 10.1093/nar/gkp552 PMC2715261

[36] Bignell GR, Huang J, Greshock J, Watt S, Butler A et al. (2004) High-resolution analysis of DNA copy number using oligonucleotide microarrays. Genome Res 14: 287-295. doi:10.1101/gr.2012304. PubMed: 14762065. 14762065 10.1101/gr.2012304 PMC327104

[37] Dutt A, Beroukhim R (2007) Single nucleotide polymorphism array analysis of cancer. Curr Opin Oncol 19: 43-49. doi:10.1097/CCO.0b013e328011a8c1. PubMed: 17133111. 17133111 10.1097/CCO.0b013e328011a8c1

[38] Jasmine F, Rahaman R, Dodsworth C, Roy S, Paul R et al. (2012) A genome-wide study of cytogenetic changes in colorectal cancer using SNP microarrays: opportunities for future personalized treatment. PLOS ONE 7: e31968. doi:10.1371/journal.pone.0031968. PubMed: 22363777. 22363777 10.1371/journal.pone.0031968 PMC3282791

[39] Rumi E, Harutyunyan A, Elena C, Pietra D, Klampfl T et al. (2011) Identification of genomic aberrations associated with disease transformation by means of high-resolution SNP array analysis in patients with myeloproliferative neoplasm. Am J Hematol 86: 974-979. doi:10.1002/ajh.22166. PubMed: 21953568. 21953568 10.1002/ajh.22166

[40] Ronchi CL, Leich E, Sbiera S, Weismann D, Rosenwald A et al. (2012) Single nucleotide polymorphism microarray analysis in cortisol-secreting adrenocortical adenomas identifies new candidate genes and pathways. Neoplasia 14: 206-218. PubMed: 22496620. 22496620 10.1593/neo.111758 PMC3323898

[41] Letouzé E, Rosati R, Komechen H, Doghman M, Marisa L et al. (2012) SNP array profiling of childhood adrenocortical tumors reveals distinct pathways of tumorigenesis and highlights candidate driver genes. J Clin Endocrinol Metab 97: E1284-E1293. doi:10.1210/jc.2012-1184. PubMed: 22539591. 22539591 10.1210/jc.2012-1184

[42] Berruti A, Baudin E, Gelderblom H, Haak HR, Porpiglia F et al. (2012) Adrenal cancer: ESMO Clinical Practice Guidelines for diagnosis, treatment and follow-up. Ann Oncol 23 Suppl 7: vii131-vii138. doi:10.1093/annonc/mds231. PubMed: 22997446. 22997446 10.1093/annonc/mds231

[43] Nieman LK, Biller BM, Findling JW, Newell-Price J, Savage MO et al. (2008) The diagnosis of Cushing’s syndrome: an Endocrine Society Clinical Practice Guideline. J Clin Endocrinol Metab 93: 1526-1540. doi:10.1210/jc.2008-0125. PubMed: 18334580. 18334580 10.1210/jc.2008-0125 PMC2386281

[44] Koschker AC, Fassnacht M, Hahner S, Weismann D, Allolio B (2006) Adrenocortical carcinoma -- improving patient care by establishing new structures. Exp Clin Endocrinol Diabetes 114: 45-51. doi:10.1055/s-2006-923808. PubMed: 16570232. 16570232 10.1055/s-2006-923808

[45] Haralambieva E, Kleiverda K, Mason DY, Schuuring E, Kluin PM (2002) Detection of three common translocation breakpoints in non-Hodgkin’s lymphomas by fluorescence in situ hybridization on routine paraffin-embedded tissue sections. J Pathol 198: 163-170. doi:10.1002/path.1197. PubMed: 12237875. 12237875 10.1002/path.1197

[46] Pfaffl MW (2001) A new mathematical model for relative quantification in real-time RT-PCR. Nucleic Acids Res 29: e45. doi:10.1093/nar/29.9.e45. PubMed: 11328886. 11328886 10.1093/nar/29.9.e45 PMC55695

[47] Nieman LK (2010) Approach to the patient with an adrenal incidentaloma. J Clin Endocrinol Metab 95: 4106-4113. doi:10.1210/jc.2010-0457. PubMed: 20823463. 20823463 10.1210/jc.2010-0457 PMC2936073

[48] Al-Sohaily S, Biankin A, Leong R, Kohonen-Corish M, Warusavitarne J (2012) Molecular pathways in colorectal cancer. J Gastroenterol Hepatol 27: 1423-1431. doi:10.1111/j.1440-1746.2012.07200.x. PubMed: 22694276. 22694276 10.1111/j.1440-1746.2012.07200.x

[49] Kang JU, Koo SH, Kwon KC, Park JW, Kim JM (2008) Gain at chromosomal region 5p15.33, containing TERT, is the most frequent genetic event in early stages of non-small cell lung cancer. Cancer Genet Cytogenet 182: 1-11. doi:10.1016/j.cancergencyto.2007.12.004. PubMed: 18328944. 18328944 10.1016/j.cancergencyto.2007.12.004

[50] Carvalho B, Postma C, Mongera S, Hopmans E, Diskin S et al. (2009) Multiple putative oncogenes at the chromosome 20q amplicon contribute to colorectal adenoma to carcinoma progression. Gut 58: 79-89. doi:10.1136/gut.2007.143065. PubMed: 18829976. 18829976 10.1136/gut.2007.143065

[51] Sillars-Hardebol AH, Carvalho B, Tijssen M, Beliën JA, de Wit M et al. (2012) TPX2 and AURKA promote 20q amplicon-driven colorectal adenoma to carcinoma progression. Gut 61: 1568-1575. doi:10.1136/gutjnl-2011-301153. PubMed: 22207630. 22207630 10.1136/gutjnl-2011-301153

[52] Buffart TE, van Grieken NC, Tijssen M, Coffa J, Ylstra B et al. (2009) High resolution analysis of DNA copy-number aberrations of chromosomes 8, 13, and 20 in gastric cancers. Virchows Arch 455: 213-223. doi:10.1007/s00428-009-0814-y. PubMed: 19697059. 19697059 10.1007/s00428-009-0814-y PMC2744787

[53] Campbell JM, Lockwood WW, Buys TP, Chari R, Coe BP et al. (2008) Integrative genomic and gene expression analysis of chromosome 7 identified novel oncogene loci in non-small cell lung cancer. Genome 51: 1032-1039. doi:10.1139/G08-086. PubMed: 19088816. 19088816 10.1139/G08-086

[54] Sinha S, Singh RK, Alam N, Roy A, Roychoudhury S et al. (2008) Alterations in candidate genes PHF2, FANCC, PTCH1 and XPA at chromosomal 9q22.3 region: pathological significance in early- and late-onset breast carcinoma. Mol Cancer 7: 84. doi:10.1186/1476-4598-7-84. PubMed: 18990233. 18990233 10.1186/1476-4598-7-84 PMC2633285

[55] Thompson PA, Brewster AM, Kim-Anh D, Baladandayuthapani V, Broom BM et al. (2011) Selective genomic copy number imbalances and probability of recurrence in early-stage breast cancer. PLOS ONE 6: e23543. doi:10.1371/journal.pone.0023543. PubMed: 21858162. 21858162 10.1371/journal.pone.0023543 PMC3155554

[56] Berthon A, Martinez A, Bertherat J, Val P (2012) Wnt/beta-catenin signalling in adrenal physiology and tumour development. Mol Cell Endocrinol 351: 87-95. doi:10.1016/j.mce.2011.09.009. PubMed: 21930188. 21930188 10.1016/j.mce.2011.09.009

[57] Demars J, Le Bouc Y, El-Osta A, Gicquel C (2011) Epigenetic and genetic mechanisms of abnormal 11p15 genomic imprinting in Silver-Russell and Beckwith-Wiedemann syndromes. Curr Med Chem 18: 1740-1750. doi:10.2174/092986711795496764. PubMed: 21466477. 21466477 10.2174/092986711795496764

[58] Bhusari S, Yang B, Kueck J, Huang W, Jarrard DF (2011) Insulin-like growth factor-2 (IGF2) loss of imprinting marks a field defect within human prostates containing cancer. Prostate 71: 1621-1630. doi:10.1002/pros.21379. PubMed: 21432864. 21432864 10.1002/pros.21379 PMC3825178

[59] Gicquel C, Raffin-Sanson ML, Gaston V, Bertagna X, Plouin PF et al. (1997) Structural and functional abnormalities at 11p15 are associated with the malignant phenotype in sporadic adrenocortical tumors: study on a series of 82 tumors. J Clin Endocrinol Metab 82: 2559-2565. doi:10.1210/jc.82.8.2559. PubMed: 9253334. 9253334 10.1210/jcem.82.8.4170

[60] Gicquel C, Bertagna X, Gaston V, Coste J, Louvel A et al. (2001) Molecular markers and long-term recurrences in a large cohort of patients with sporadic adrenocortical tumors. Cancer Res 61: 6762-6767. PubMed: 11559548. 11559548

[61] Giordano TJ, Kuick R, Else T, Gauger PG, Vinco M et al. (2009) Molecular classification and prognostication of adrenocortical tumors by transcriptome profiling. Clin Cancer Res 15: 668-676. doi:10.1158/1078-0432.CCR-08-1067. PubMed: 19147773. 19147773 10.1158/1078-0432.CCR-08-1067 PMC2629378

[62] Drelon C, Berthon A, Ragazzon B, Tissier F, Bandiera R et al. (2012) Analysis of the role of Igf2 in adrenal tumour development in transgenic mouse models. PLOS ONE 7: e44171. doi:10.1371/journal.pone.0044171. PubMed: 22952916. 22952916 10.1371/journal.pone.0044171 PMC3429465

[63] Scotto L, Narayan G, Nandula SV, Subramaniyam S, Kaufmann AM et al. (2008) Integrative genomics analysis of chromosome 5p gain in cervical cancer reveals target over-expressed genes, including Drosha. Mol Cancer 7: 58. doi:10.1186/1476-4598-7-58. PubMed: 18559093. 18559093 10.1186/1476-4598-7-58 PMC2440550

[64] Barreau O, de Reynies A, Wilmot-Roussel H, Guillaud-Bataille M, Auzan C et al. (2012) Clinical and pathophysiological implications of chromosomal alterations in adrenocortical tumors: an integrated genomic approach. J Clin Endocrinol Metab 97: E301-E311. doi:10.1210/jc.2011-1588. PubMed: 22112813. 22112813 10.1210/jc.2011-1588

