# Supplementary figures and images for: Single Nucleotide Polymorphism Array Profiling of Adrenocortical Tumors - Evidence for an Adenoma Carcinoma Sequence?
Source: PLoS One. 2013 Sep 16;8(9):e73959. doi: 10.1371/journal.pone.0073959 (PMC3774745; doi:10.1371/journal.pone.0073959)

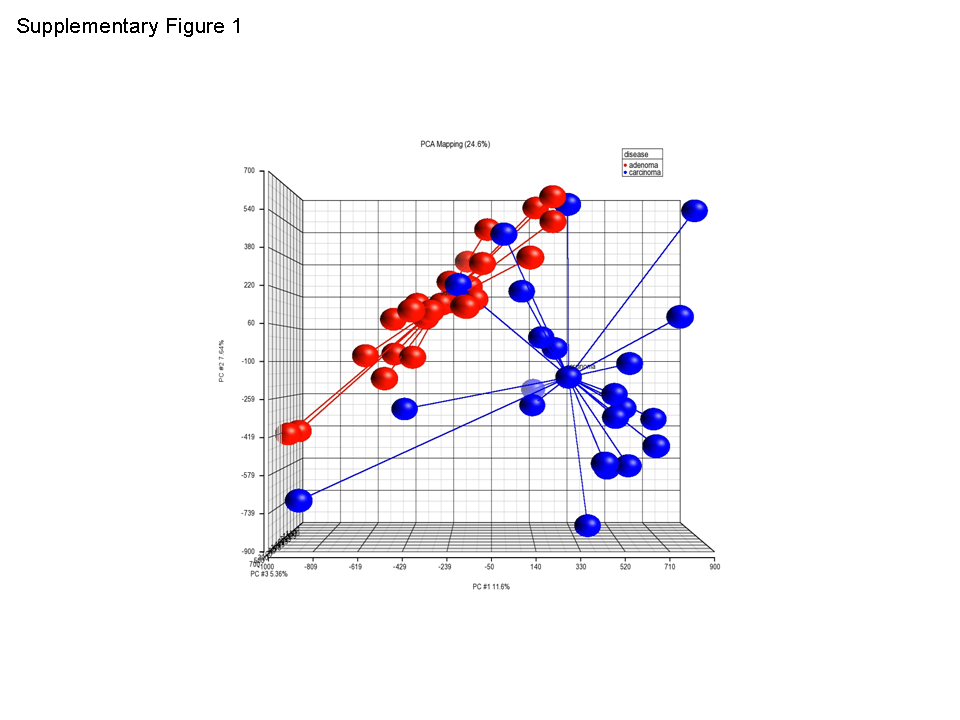

Supplement: Figure S1 — Principal component analysis for the SNP array analysis of 46 adrenocortical tumors (24 adenoma in blue and 22 carcinoma in red) by Partek Genomic Suite Software. (TIF) [file pone.0073959.s001.tif]
