# Supplementary material for: Single Nucleotide Polymorphism Array Profiling of Adrenocortical Tumors - Evidence for an Adenoma Carcinoma Sequence?
Source: PLoS One. 2013 Sep 16;8(9):e73959. doi: 10.1371/journal.pone.0073959 (PMC3774745; doi:10.1371/journal.pone.0073959)
Supplement: Figure S2 — Enrichment analysis including the genes with copy number alterations observed only in carcinomas (recurrent CNA, in at least 4 samples). A) Analysis generated including genes affected by copy number gains (n=11414). B) Analysis generated including genes affected by copy number gains (n=3717). Gene family analysis by GSEA; pathway and gene process analysis by GeneGo (Meta Core Analytical suite, P values expresses as logarithmic scale; detailed legend available at http://pathwaymaps.com/pdf/MC_legend.pdf.). (PPT) [file pone.0073959.s002.ppt]

## Slide 1
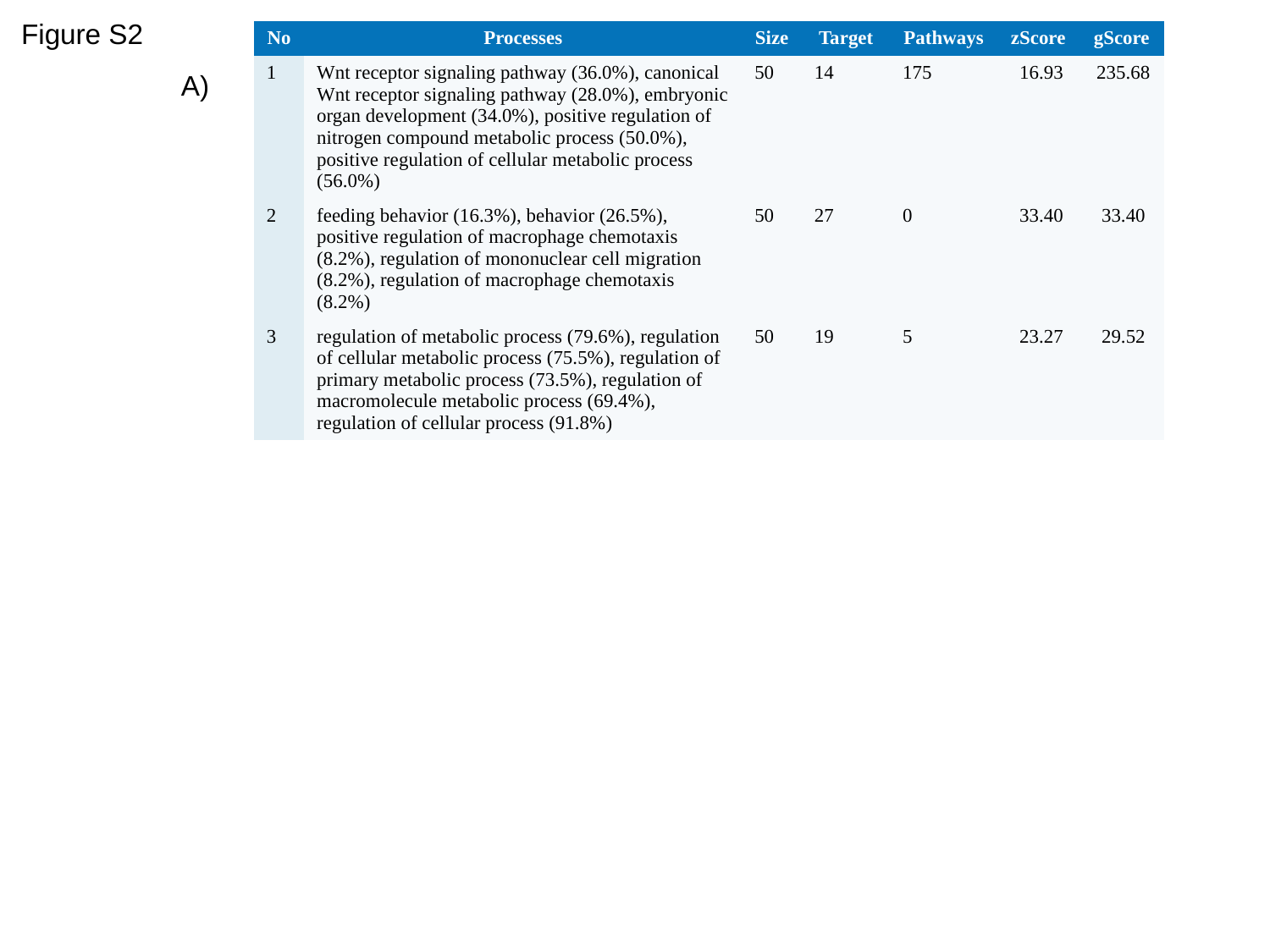

Figure S2
| No | Processes | Size | Target | Pathways | zScore | gScore |
| --- | --- | --- | --- | --- | --- | --- |
| 1 | Wnt receptor signaling pathway (36.0%), canonical Wnt receptor signaling pathway (28.0%), embryonic organ development (34.0%), positive regulation of nitrogen compound metabolic process (50.0%), positive regulation of cellular metabolic process (56.0%) | 50 | 14 | 175 | 16.93 | 235.68 |
| 2 | feeding behavior (16.3%), behavior (26.5%), positive regulation of macrophage chemotaxis (8.2%), regulation of mononuclear cell migration (8.2%), regulation of macrophage chemotaxis (8.2%) | 50 | 27 | 0 | 33.40 | 33.40 |
| 3 | regulation of metabolic process (79.6%), regulation of cellular metabolic process (75.5%), regulation of primary metabolic process (73.5%), regulation of macromolecule metabolic process (69.4%), regulation of cellular process (91.8%) | 50 | 19 | 5 | 23.27 | 29.52 |
A)

## Slide 2
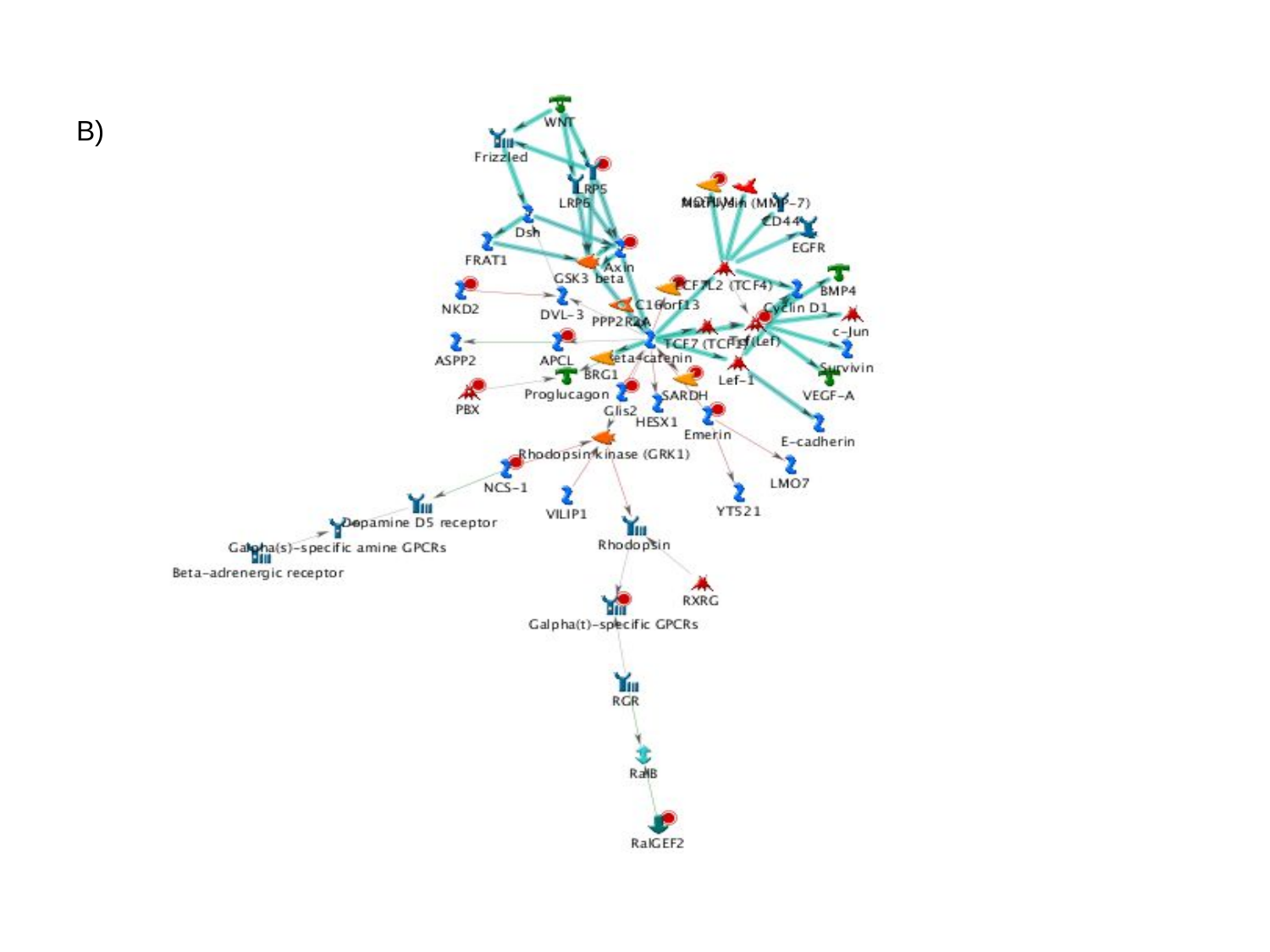

B)
