# Supplementary material for: Single Nucleotide Polymorphism Array Profiling of Adrenocortical Tumors - Evidence for an Adenoma Carcinoma Sequence?
Source: PLoS One. 2013 Sep 16;8(9):e73959. doi: 10.1371/journal.pone.0073959 (PMC3774745; doi:10.1371/journal.pone.0073959)
Supplement: Figure S3 — A) Results of the generation of biological networks using Analyze Networks (AN) algorithm with default settings. The gene content of the uploaded files (genes with LOH events observed in at least 4 samples in carcinomas, n=11415) is used as the input list. This is a variant of the shortest paths algorithm with main parameters of 1. relative enrichment with the uploaded data, and 2. relative saturation of networks with canonical pathways. In this workflow the networks are prioritized based on the number of fragments of canonical pathways on the network. B) Graphical representation of the top scored (by the number of pathways) analysed network (positive regulation of the macromolecule metabolic process). Thick cyan lines indicate the fragments of canonical pathways. Up-regulated genes are marked with red circles; down-regulated with blue circles. The 'checkerboard' color indicates mixed expression for the gene between files or between multiple tags for the same gene. Detailed legend is available at http://pathwaymaps.com/pdf/MC_legend.pdf. (PPT) [file pone.0073959.s003.ppt]

## Slide 1
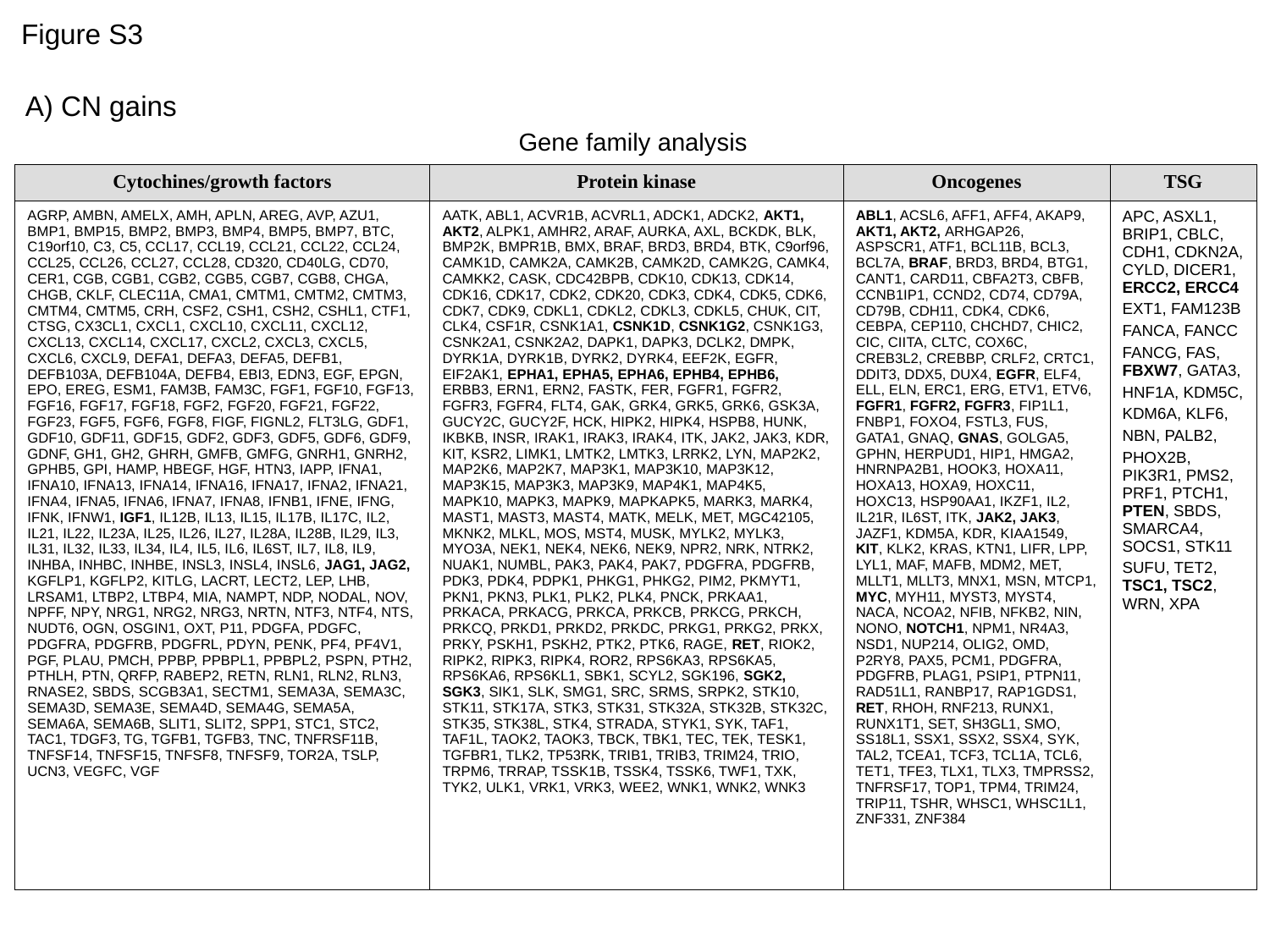

Figure S3
A) CN gains
Gene family analysis
| Cytochines/growth factors | Protein kinase | Oncogenes | TSG |
| --- | --- | --- | --- |
| AGRP, AMBN, AMELX, AMH, APLN, AREG, AVP, AZU1, BMP1, BMP15, BMP2, BMP3, BMP4, BMP5, BMP7, BTC, C19orf10, C3, C5, CCL17, CCL19, CCL21, CCL22, CCL24, CCL25, CCL26, CCL27, CCL28, CD320, CD40LG, CD70, CER1, CGB, CGB1, CGB2, CGB5, CGB7, CGB8, CHGA, CHGB, CKLF, CLEC11A, CMA1, CMTM1, CMTM2, CMTM3, CMTM4, CMTM5, CRH, CSF2, CSH1, CSH2, CSHL1, CTF1, CTSG, CX3CL1, CXCL1, CXCL10, CXCL11, CXCL12, CXCL13, CXCL14, CXCL17, CXCL2, CXCL3, CXCL5, CXCL6, CXCL9, DEFA1, DEFA3, DEFA5, DEFB1, DEFB103A, DEFB104A, DEFB4, EBI3, EDN3, EGF, EPGN, EPO, EREG, ESM1, FAM3B, FAM3C, FGF1, FGF10, FGF13, FGF16, FGF17, FGF18, FGF2, FGF20, FGF21, FGF22, FGF23, FGF5, FGF6, FGF8, FIGF, FIGNL2, FLT3LG, GDF1, GDF10, GDF11, GDF15, GDF2, GDF3, GDF5, GDF6, GDF9, GDNF, GH1, GH2, GHRH, GMFB, GMFG, GNRH1, GNRH2, GPHB5, GPI, HAMP, HBEGF, HGF, HTN3, IAPP, IFNA1, IFNA10, IFNA13, IFNA14, IFNA16, IFNA17, IFNA2, IFNA21, IFNA4, IFNA5, IFNA6, IFNA7, IFNA8, IFNB1, IFNE, IFNG, IFNK, IFNW1, IGF1, IL12B, IL13, IL15, IL17B, IL17C, IL2, IL21, IL22, IL23A, IL25, IL26, IL27, IL28A, IL28B, IL29, IL3, IL31, IL32, IL33, IL34, IL4, IL5, IL6, IL6ST, IL7, IL8, IL9, INHBA, INHBC, INHBE, INSL3, INSL4, INSL6, JAG1, JAG2, KGFLP1, KGFLP2, KITLG, LACRT, LECT2, LEP, LHB, LRSAM1, LTBP2, LTBP4, MIA, NAMPT, NDP, NODAL, NOV, NPFF, NPY, NRG1, NRG2, NRG3, NRTN, NTF3, NTF4, NTS, NUDT6, OGN, OSGIN1, OXT, P11, PDGFA, PDGFC, PDGFRA, PDGFRB, PDGFRL, PDYN, PENK, PF4, PF4V1, PGF, PLAU, PMCH, PPBP, PPBPL1, PPBPL2, PSPN, PTH2, PTHLH, PTN, QRFP, RABEP2, RETN, RLN1, RLN2, RLN3, RNASE2, SBDS, SCGB3A1, SECTM1, SEMA3A, SEMA3C, SEMA3D, SEMA3E, SEMA4D, SEMA4G, SEMA5A, SEMA6A, SEMA6B, SLIT1, SLIT2, SPP1, STC1, STC2, TAC1, TDGF3, TG, TGFB1, TGFB3, TNC, TNFRSF11B, TNFSF14, TNFSF15, TNFSF8, TNFSF9, TOR2A, TSLP, UCN3, VEGFC, VGF | AATK, ABL1, ACVR1B, ACVRL1, ADCK1, ADCK2, AKT1, AKT2, ALPK1, AMHR2, ARAF, AURKA, AXL, BCKDK, BLK, BMP2K, BMPR1B, BMX, BRAF, BRD3, BRD4, BTK, C9orf96, CAMK1D, CAMK2A, CAMK2B, CAMK2D, CAMK2G, CAMK4, CAMKK2, CASK, CDC42BPB, CDK10, CDK13, CDK14, CDK16, CDK17, CDK2, CDK20, CDK3, CDK4, CDK5, CDK6, CDK7, CDK9, CDKL1, CDKL2, CDKL3, CDKL5, CHUK, CIT, CLK4, CSF1R, CSNK1A1, CSNK1D, CSNK1G2, CSNK1G3, CSNK2A1, CSNK2A2, DAPK1, DAPK3, DCLK2, DMPK, DYRK1A, DYRK1B, DYRK2, DYRK4, EEF2K, EGFR, EIF2AK1, EPHA1, EPHA5, EPHA6, EPHB4, EPHB6, ERBB3, ERN1, ERN2, FASTK, FER, FGFR1, FGFR2, FGFR3, FGFR4, FLT4, GAK, GRK4, GRK5, GRK6, GSK3A, GUCY2C, GUCY2F, HCK, HIPK2, HIPK4, HSPB8, HUNK, IKBKB, INSR, IRAK1, IRAK3, IRAK4, ITK, JAK2, JAK3, KDR, KIT, KSR2, LIMK1, LMTK2, LMTK3, LRRK2, LYN, MAP2K2, MAP2K6, MAP2K7, MAP3K1, MAP3K10, MAP3K12, MAP3K15, MAP3K3, MAP3K9, MAP4K1, MAP4K5, MAPK10, MAPK3, MAPK9, MAPKAPK5, MARK3, MARK4, MAST1, MAST3, MAST4, MATK, MELK, MET, MGC42105, MKNK2, MLKL, MOS, MST4, MUSK, MYLK2, MYLK3, MYO3A, NEK1, NEK4, NEK6, NEK9, NPR2, NRK, NTRK2, NUAK1, NUMBL, PAK3, PAK4, PAK7, PDGFRA, PDGFRB, PDK3, PDK4, PDPK1, PHKG1, PHKG2, PIM2, PKMYT1, PKN1, PKN3, PLK1, PLK2, PLK4, PNCK, PRKAA1, PRKACA, PRKACG, PRKCA, PRKCB, PRKCG, PRKCH, PRKCQ, PRKD1, PRKD2, PRKDC, PRKG1, PRKG2, PRKX, PRKY, PSKH1, PSKH2, PTK2, PTK6, RAGE, RET, RIOK2, RIPK2, RIPK3, RIPK4, ROR2, RPS6KA3, RPS6KA5, RPS6KA6, RPS6KL1, SBK1, SCYL2, SGK196, SGK2, SGK3, SIK1, SLK, SMG1, SRC, SRMS, SRPK2, STK10, STK11, STK17A, STK3, STK31, STK32A, STK32B, STK32C, STK35, STK38L, STK4, STRADA, STYK1, SYK, TAF1, TAF1L, TAOK2, TAOK3, TBCK, TBK1, TEC, TEK, TESK1, TGFBR1, TLK2, TP53RK, TRIB1, TRIB3, TRIM24, TRIO, TRPM6, TRRAP, TSSK1B, TSSK4, TSSK6, TWF1, TXK, TYK2, ULK1, VRK1, VRK3, WEE2, WNK1, WNK2, WNK3 | ABL1, ACSL6, AFF1, AFF4, AKAP9, AKT1, AKT2, ARHGAP26, ASPSCR1, ATF1, BCL11B, BCL3, BCL7A, BRAF, BRD3, BRD4, BTG1, CANT1, CARD11, CBFA2T3, CBFB, CCNB1IP1, CCND2, CD74, CD79A, CD79B, CDH11, CDK4, CDK6, CEBPA, CEP110, CHCHD7, CHIC2, CIC, CIITA, CLTC, COX6C, CREB3L2, CREBBP, CRLF2, CRTC1, DDIT3, DDX5, DUX4, EGFR, ELF4, ELL, ELN, ERC1, ERG, ETV1, ETV6, FGFR1, FGFR2, FGFR3, FIP1L1, FNBP1, FOXO4, FSTL3, FUS, GATA1, GNAQ, GNAS, GOLGA5, GPHN, HERPUD1, HIP1, HMGA2, HNRNPA2B1, HOOK3, HOXA11, HOXA13, HOXA9, HOXC11, HOXC13, HSP90AA1, IKZF1, IL2, IL21R, IL6ST, ITK, JAK2, JAK3, JAZF1, KDM5A, KDR, KIAA1549, KIT, KLK2, KRAS, KTN1, LIFR, LPP, LYL1, MAF, MAFB, MDM2, MET, MLLT1, MLLT3, MNX1, MSN, MTCP1, MYC, MYH11, MYST3, MYST4, NACA, NCOA2, NFIB, NFKB2, NIN, NONO, NOTCH1, NPM1, NR4A3, NSD1, NUP214, OLIG2, OMD, P2RY8, PAX5, PCM1, PDGFRA, PDGFRB, PLAG1, PSIP1, PTPN11, RAD51L1, RANBP17, RAP1GDS1, RET, RHOH, RNF213, RUNX1, RUNX1T1, SET, SH3GL1, SMO, SS18L1, SSX1, SSX2, SSX4, SYK, TAL2, TCEA1, TCF3, TCL1A, TCL6, TET1, TFE3, TLX1, TLX3, TMPRSS2, TNFRSF17, TOP1, TPM4, TRIM24, TRIP11, TSHR, WHSC1, WHSC1L1, ZNF331, ZNF384 | APC, ASXL1, BRIP1, CBLC, CDH1, CDKN2A, CYLD, DICER1, ERCC2, ERCC4 EXT1, FAM123B FANCA, FANCC FANCG, FAS, FBXW7, GATA3, HNF1A, KDM5C, KDM6A, KLF6, NBN, PALB2, PHOX2B, PIK3R1, PMS2, PRF1, PTCH1, PTEN, SBDS, SMARCA4, SOCS1, STK11 SUFU, TET2, TSC1, TSC2, WRN, XPA |

## Slide 2
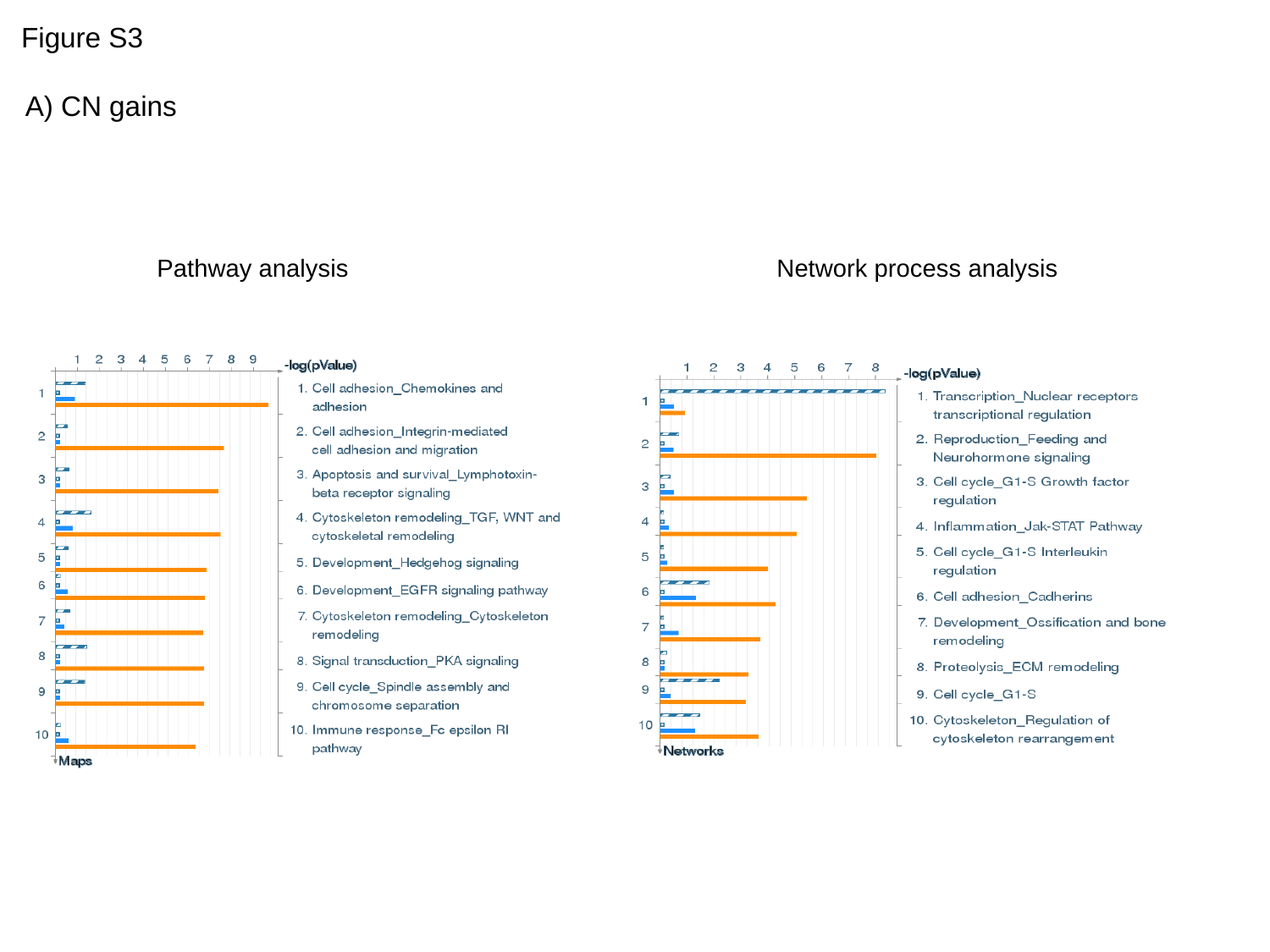

Figure S3
A) CN gains
Pathway analysis
Network process analysis

## Slide 3
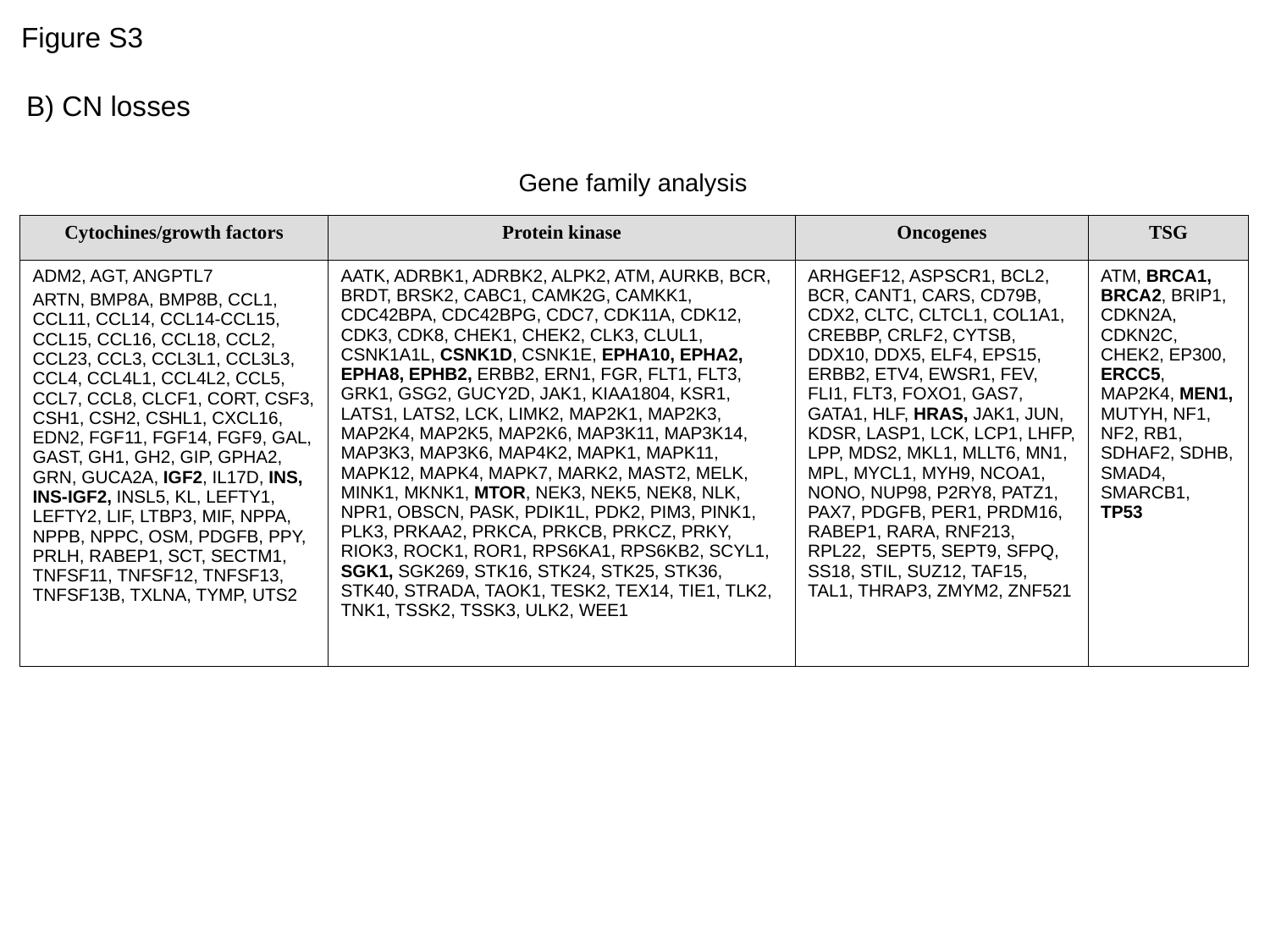

Figure S3
B) CN losses
Gene family analysis
| Cytochines/growth factors | Protein kinase | Oncogenes | TSG |
| --- | --- | --- | --- |
| ADM2, AGT, ANGPTL7 ARTN, BMP8A, BMP8B, CCL1, CCL11, CCL14, CCL14-CCL15, CCL15, CCL16, CCL18, CCL2, CCL23, CCL3, CCL3L1, CCL3L3, CCL4, CCL4L1, CCL4L2, CCL5, CCL7, CCL8, CLCF1, CORT, CSF3, CSH1, CSH2, CSHL1, CXCL16, EDN2, FGF11, FGF14, FGF9, GAL, GAST, GH1, GH2, GIP, GPHA2, GRN, GUCA2A, IGF2, IL17D, INS, INS-IGF2, INSL5, KL, LEFTY1, LEFTY2, LIF, LTBP3, MIF, NPPA, NPPB, NPPC, OSM, PDGFB, PPY, PRLH, RABEP1, SCT, SECTM1, TNFSF11, TNFSF12, TNFSF13, TNFSF13B, TXLNA, TYMP, UTS2 | AATK, ADRBK1, ADRBK2, ALPK2, ATM, AURKB, BCR, BRDT, BRSK2, CABC1, CAMK2G, CAMKK1, CDC42BPA, CDC42BPG, CDC7, CDK11A, CDK12, CDK3, CDK8, CHEK1, CHEK2, CLK3, CLUL1, CSNK1A1L, CSNK1D, CSNK1E, EPHA10, EPHA2, EPHA8, EPHB2, ERBB2, ERN1, FGR, FLT1, FLT3, GRK1, GSG2, GUCY2D, JAK1, KIAA1804, KSR1, LATS1, LATS2, LCK, LIMK2, MAP2K1, MAP2K3, MAP2K4, MAP2K5, MAP2K6, MAP3K11, MAP3K14, MAP3K3, MAP3K6, MAP4K2, MAPK1, MAPK11, MAPK12, MAPK4, MAPK7, MARK2, MAST2, MELK, MINK1, MKNK1, MTOR, NEK3, NEK5, NEK8, NLK, NPR1, OBSCN, PASK, PDIK1L, PDK2, PIM3, PINK1, PLK3, PRKAA2, PRKCA, PRKCB, PRKCZ, PRKY, RIOK3, ROCK1, ROR1, RPS6KA1, RPS6KB2, SCYL1, SGK1, SGK269, STK16, STK24, STK25, STK36, STK40, STRADA, TAOK1, TESK2, TEX14, TIE1, TLK2, TNK1, TSSK2, TSSK3, ULK2, WEE1 | ARHGEF12, ASPSCR1, BCL2, BCR, CANT1, CARS, CD79B, CDX2, CLTC, CLTCL1, COL1A1, CREBBP, CRLF2, CYTSB, DDX10, DDX5, ELF4, EPS15, ERBB2, ETV4, EWSR1, FEV, FLI1, FLT3, FOXO1, GAS7, GATA1, HLF, HRAS, JAK1, JUN, KDSR, LASP1, LCK, LCP1, LHFP, LPP, MDS2, MKL1, MLLT6, MN1, MPL, MYCL1, MYH9, NCOA1, NONO, NUP98, P2RY8, PATZ1, PAX7, PDGFB, PER1, PRDM16, RABEP1, RARA, RNF213, RPL22, SEPT5, SEPT9, SFPQ, SS18, STIL, SUZ12, TAF15, TAL1, THRAP3, ZMYM2, ZNF521 | ATM, BRCA1, BRCA2, BRIP1, CDKN2A, CDKN2C, CHEK2, EP300, ERCC5, MAP2K4, MEN1, MUTYH, NF1, NF2, RB1, SDHAF2, SDHB, SMAD4, SMARCB1, TP53 |

## Slide 4
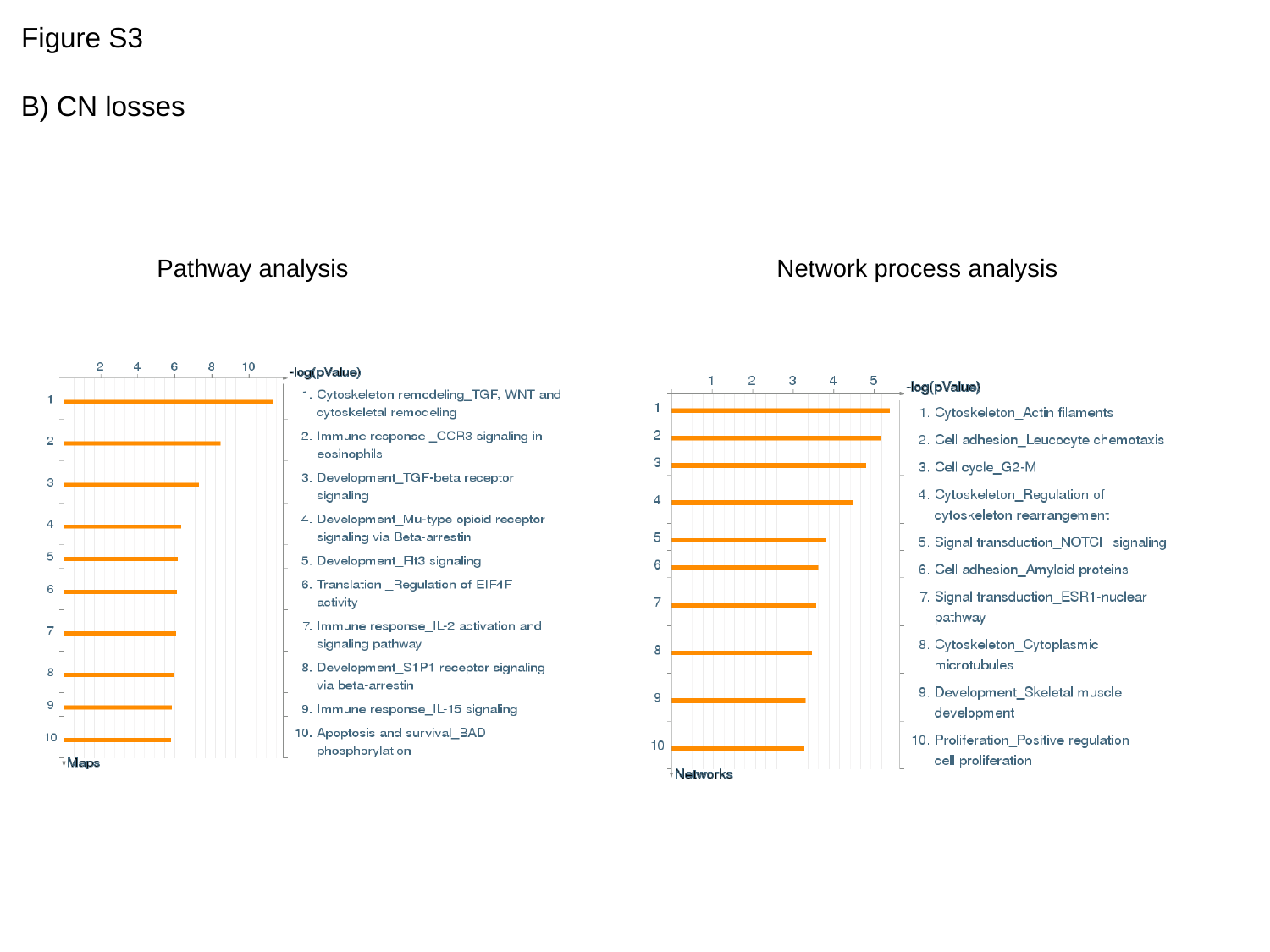

Figure S3
B) CN losses
Pathway analysis
Network process analysis
