# Supplementary material for: Single Nucleotide Polymorphism Array Profiling of Adrenocortical Tumors - Evidence for an Adenoma Carcinoma Sequence?
Source: PLoS One. 2013 Sep 16;8(9):e73959. doi: 10.1371/journal.pone.0073959 (PMC3774745; doi:10.1371/journal.pone.0073959)
Supplement: Table S5 — Gene family analysis (GSEA) of all the most frequently altered genes observed at the SNP array analysis in adrenocortical tumors, subdivided into adenomas and carcinomas. (DOC) [file pone.0073959.s008.doc]

Table S5.

| **Cytochines and growth factors** | **Transcription factors** | **Protein kinase** | **Oncogenes** | **TSG** |
| --- | --- | --- | --- | --- |
| **Adenoma – Frequent CN gains (n= 4/24)** | | | | |
| AMH, AZU1, FGF22, **IGF2, INS,** INS-IGF2, LRSAM1, PDGFA, QRFP, SCT, SECTM1, SEMA4A, SLURP1, TNC, TNFSF15, TNFSF8, TOR2A | ARC, ARID3A, ASCL2, BARHL1, BRD3, C19orf6, DEAF1, DIDO1, **E4F1**, FAM20C, FOXH1, FOXK2, GFI1B, GTF3C4, GTF3C5, **HES4, HES5**, HSF1, IRF7, KLF16, LHX2, LHX3, LHX6, LRRC14, MAFG, MAFK, MECP2, MED16, MED27, MFSD3, MYT1, NFKBIL2, **NR5A1**, NR6A1, PBX3, PHRF1, PRDM12, PRDM16, PRDM7, PRRX2, RASSF7, RXRA, SCRT1, SFRS17A, SHOX, SNAPC4, SOX18, SOX8, TCF3, TCFL5, THOC4, TRIP13, WDR45L, ZBTB6, ZFP37, ZNF16, ZNF250, ZNF251, ZNF696, ZNF7, ZNF79 | AATK, ABL1, ADCK5, BRD3, BRSK2, C9orf96, CDK10, CDK11A, CDK9, **CSNK1D, CSNK1G2,** IRAK1, MAPK15, MKNK2, NEK6, NRBP2, PKN3, PRKCZ, PTK2, PTK6, SRMS, STK11, WNK2 | ABL1, ASPSCR1, BRD3, CRLF2, FNBP1, FSTL3, **HRAS, NOTCH1,** NUP214, P2RY8, PRDM16, SET, TCF3 | FANCA, RECQL4, STK11, **TSC1, TSC2** |
| **Carcinoma – Frequent CN gains (n=4/22)** | | | | |
| AGRP, AMBN, AMELX, AMH, APLN, AREG, AVP, AZU1, BMP1, BMP15, BMP2, BMP3, BMP4, BMP5, BMP7, BTC, C19orf10, C3, C5, CCL17, CCL19, CCL21, CCL22, CCL24, CCL25, CCL26, CCL27, CCL28, CD320, CD40LG, CD70, CER1, CGB, CGB1, CGB2, CGB5, CGB7, CGB8, CHGA, CHGB, CKLF, CLEC11A, CMA1, CMTM1, CMTM2, CMTM3, CMTM4, CMTM5, CRH, CSF2, CSH1, CSH2, CSHL1, CTF1, CTSG, CX3CL1, CXCL1, CXCL10, CXCL11, CXCL12, CXCL13, CXCL14, CXCL17, CXCL2, CXCL3, CXCL5, CXCL6, CXCL9, DEFA1, DEFA3, DEFA5, DEFB1, DEFB103A, DEFB104A, DEFB4, EBI3, EDN3, EGF, EPGN, EPO, EREG, ESM1, FAM3B, FAM3C, FGF1, FGF10, FGF13, FGF16, FGF17, FGF18, FGF2, FGF20, FGF21, FGF22, FGF23, FGF5, FGF6, FGF8, FIGF, FIGNL2, FLT3LG, GDF1, GDF10, GDF11, GDF15, GDF2, GDF3, GDF5, GDF6, GDF9, GDNF, GH1, GH2, GHRH, GMFB, GMFG, GNRH1, GNRH2, GPHB5, GPI, HAMP, HBEGF, HGF, HTN3, IAPP, IFNA1, IFNA10, IFNA13, IFNA14, IFNA16, IFNA17, IFNA2, IFNA21, IFNA4, IFNA5, IFNA6, IFNA7, IFNA8, IFNB1, IFNE, IFNG, IFNK, IFNW1, **IGF1**, IL12B, IL13, IL15, IL17B, IL17C, IL2, IL21, IL22, IL23A, IL25, IL26, IL27, IL28A, IL28B, IL29, IL3, IL31, IL32, IL33, IL34, IL4, IL5, IL6, IL6ST, IL7, IL8, IL9, INHBA, INHBC, INHBE, INSL3, INSL4, INSL6, **JAG1, JAG2,** KGFLP1, KGFLP2, KITLG, LACRT, LECT2, LEP, LHB, LRSAM1, LTBP2, LTBP4, MIA, NAMPT, NDP, NODAL, NOV, NPFF, NPY, NRG1, NRG2, NRG3, NRTN, NTF3, NTF4, NTS, NUDT6, OGN, OSGIN1, OXT, P11, PDGFA, PDGFC, PDGFRA, PDGFRB, PDGFRL, PDYN, PENK, PF4, PF4V1, PGF, PLAU, PMCH, PPBP, PPBPL1, PPBPL2, PSPN, PTH2, PTHLH, PTN, QRFP, RABEP2, RETN, RLN1, RLN2, RLN3, RNASE2, SBDS, SCGB3A1, SECTM1, SEMA3A, SEMA3C, SEMA3D, SEMA3E, SEMA4D, SEMA4G, SEMA5A, SEMA6A, SEMA6B, SLIT1, SLIT2, SPP1, STC1, STC2, TAC1, TDGF3, TG, TGFB1, TGFB3, TNC, TNFRSF11B, TNFSF14, TNFSF15, TNFSF8, TNFSF9, TOR2A, TSLP, UCN3, VEGFC, VGF | ADNP, AFF1, AFF2, AFF4, AHR, **AIRE**, ALX1, AR, ARID3A, ASCL1, ASH2L, ATF1, ATF5, ATF7, ATF7IP, ATN1, ATOH1, ATOH7, ATRX, ATXN2, BACH1, BANP, BARHL1, BARX1, BATF, BAZ1A, BAZ1B, BAZ2A, BCL11B, BCL3, BHLHE22, BHLHE41, BLOC1S1, BNC2, BPTF, BRD3, BRD4, BRD7, BRF1, BTAF1, BTBD3, BTF3, C14orf101, C14orf106, C14orf166, C16orf80, C19orf28, C19orf6, CAND1, CASK, CBFA2T3, CBX2, CBX3, CBX4, CBX5, CBX8, CDK7, CDR2, CDX1, CDX4, CEBPA, CEBPB, CEBPD, CEBPE, CEBPG, CHD1, CHD4, CHD6, CIC, CIITA, CITED1, CLOCK, CNOT4, CNOT8, COPS5, CORO1A, CREB3, CREB5, CREBBP, CREBL2, CRIP1, CRIP2, CRX, CSDA, CSRP2, CTCF, CUX1, CUX2, DBP, DDIT3, DIDO1, DLX5, DLX6, DMRT1, DMRT2, DMRTC1, DMTF1, DPF1, DPF3, DTX2, DUX4, E2F1, E2F4, E2F5, **E4F1**, EBF1, EBF4, ECD, EGLN2, EGR1, EGR3, ELF2, ELF4, ELK1, ELK3, EMX2, EN2, EP400, **ERCC2, ERCC6**, ERF, ERG, ERGIC2, ERMP1, ESR2, ESRRB, ETS2, ETV1, ETV2, ETV6, EVX1, EYA1, EYA2, EZH2, FAM20C, FEM1A, FHL1, FOS, FOSB, FOXA1, FOXA2, FOXA3, FOXC2, FOXD1, FOXD4, FOXE1, FOXF1, FOXG1, FOXI1, FOXJ1, FOXJ2, FOXK2, FOXL1, FOXM1, FOXN3, FOXO4, FOXP2, FOXP3, FOXS1, GABPA, GATA1, GATA3, GATA4, GATA5, GBX1, GFI1B, GLI1, GLI3, GLIS2, GRLF1, GSC, GSX2, GTF2A1, GTF2F1, GTF2H2, GTF2H3, GTF2I, GTF3C1, GTF3C4, GTF3C5, HAND1, HAND2, HCFC2, HEY1, HHEX, HIF1A, HIF3A, HKR1, HMG20B, HMGA2, HMGB2, HMGXB3, HMX2, HMX3, HNF1A, HNF4A, HNF4G, HOPX, HOXA1, HOXA10, HOXA11, HOXA13, HOXA2, HOXA3, HOXA4, HOXA5, HOXA6, HOXA7, HOXA9, HOXC10, HOXC11, HOXC12, HOXC13, HOXC4, HOXC5, HOXC6, HOXC8, HOXC9, HR, HSF2BP, HSF4, HSFX1, HSFY1, ID1, IER2, IKZF1, IKZF5, ILF3, INSM1, INSM2, IRF1, IRF2, IRF3, IRF5, IRF8, IRF9, IRX1, IRX2, IRX3, IRX4, IRX5, IRX6, ISL1, JUNB, JUND, KDM3B, KDM4C, KDM5C, KEAP1, KLF1, KLF10, KLF14, KLF16, KLF2, KLF3, KLF4, KLF6, KLF8, KLF9, KLHL5, L3MBTL, LARP1, LAS1L, LBX1, LDB1, LDB2, LDOC1, **LEF1,** LHX2, LHX3, LHX5, LHX6, LIMA1, LITAF, LMO3, LMX1B, LOC729991-MEF2B, LSR, LYL1, LZTS1, MAF, MAFB, MAFG, MAFK, MAMLD1, MANSC1, MAX, MAZ, MECP2, MED12, MED14, MED16, MED21, MED26, MED27, MED6, MED7, MEF2C, MEFV, MEIS3, MEOX2, METTL3, MID1, MLL2, MLL4, MLLT1, MLLT3, MLXIP, MLXIPL, MNAT1, MNX1, MORF4, MORF4L2, MSC, MSX1, MSX2, MTA1, MXD3, MXD4, MXI1, MYBL1, MYBL2, MYC, MYF5, MYF6, MYST3, MYST4, MYT1, NAB2, NANOG, NCALD, NCOA2, NCOA3, NCOA6, NCOR2, NEUROD4, NEUROD6, NEUROG1, NEUROG2, NEUROG3, NFAT5, NFATC2, NFATC3, NFATC4, NFE2, NFE2L3, NFIB, NFIC, NFIL3, NFIX, NFKB1, NFKB2, NFKBIA, NFKBIB, NFX1, NFYB, NKRF, NKX2-1, NKX2-2, NKX2-3, NKX2-4, NKX2-5, NKX2-8, NKX3-1, NKX3-2, NKX6-1, NKX6-2, NOBOX, NPAS1, NR0B1, NR1H2, NR1H4, NR2C1, NR2F1, NR2F6, NR3C1, NR3C2, NR4A1, NR4A3, **NR5A1**, NR6A1, NRF1, NRIP1, NRL, OLIG1, OLIG2, OPTN, OTX2, PACS2, PAX1, PAX2, PAX4, PAX5, PAX9, PAXIP1, PBX3, PBX4, PCGF3, PDLIM5, PFDN5, PHC1, PHOX2B, PITX1, PITX2, PITX3, PKNOX1, PLAG1, PLAGL2, PLRG1, POU2F2, POU3F4, POU4F2, POU4F3, POU6F1, POU6F2, PPARGC1A, PPP1R13L, PRDM12, PRDM14, PRDM15, PRDM4, PRDM5, PRDM6, PRDM7, PRDM8, PRDM9, PRICKLE3, PROP1, PRR7, PRRX2, PSIP1, PSMC5, PTTG1IP, PURA, RARG, RBBP9, RBL1, RBL2, RBPJ, RBPJL, RCOR1, RELB, REPIN1, REST, RFX1, RFX2, RFX3, RFX4, RFXANK, RLIM, RNF10, RNF113A, RNF114, RNF14, RNF160, RNF24, RNF4, RNF41, RORB, RRN3, RUNX1, RUNX1T1, RXRA, SAFB, SALL1, SALL2, SALL4, SAMD4B, SAP30, SAP30BP, SART3, SCAND1, SCML1, SCML2, SCRT2, SERTAD1, SETD4, SFRS17A, SHOX, SIAH1, SIM2, SIN3B, SIX1, SIX4, SIX5, SIX6, SLC25A40, SMAD1, SMAD5, SMARCA1, SMARCA2, SMARCA4, SMARCA5, SMARCAD1, SMARCC2, SNAI1, SNAI2, SNAI3, SNAPC1, SNAPC2, SNAPC3, SNAPC4, SND1, SNW1, SOX12, SOX17, SOX18, SOX3, SOX30, SOX5, SOX8, SOX9, SP1, SP4, SPIB, SRA1, SRCAP, SRY, SSX1, SSX2, SSX3, SSX4, SSX5, SSX6, SSX7, SSX8, SSX9, ST18, STAT2, STAT6, SUB1, SUPT16H, SUPT5H, TAF1, TAF1C, TAF2, TAF3, TAF4, TAF5, TAF6, TAF7, TAF9, TAL2, TBC1D10B, TBL1Y, TBX2, TBX20, TBX22, TBX3, TBX4, TBX5, TBX6, TCEAL1, TCERG1, TCF15, TCF3, TCF7, TCF7L2, TCFL5, TEAD2, TEAD4, TECPR1, TFAM, TFAP2C, TFAP4, TFCP2, TFDP3, TFE3, TFEC, TGFB1I1, THOC4, TIMELESS, TLX1, TLX3, TNRC6A, TOX, TOX3, TRIM24, TRIP10, TRIP11, TRIP13, TRIP6, TRMT1, TRPS1, TSC22D3, TSC22D4, TTLL5, TULP2, TULP3, TWIST1, UHRF1, USE1, USF2, UTF1, VAX1, VDR, VENTX, VGLL1, VSX1, WBP5, WDR45L, WHSC1, YAF2, YEATS4, YY1, ZAR1, ZBTB25, ZBTB32, ZBTB33, ZBTB39, ZBTB43, ZBTB6, ZBTB7A, ZDHHC1, ZDHHC17, ZFAND5, ZFHX3, ZFP112, ZFP30, ZFP36, ZFP37, ZFP62, ZFP64, ZFP92, ZFPM1, ZFPM2, ZFR, ZFX, ZFY, ZHX1, ZHX3, ZIC3, ZKSCAN1, ZKSCAN5, ZMYM3, ZNF10, ZNF107, ZNF117, ZNF12, ZNF131, ZNF133, ZNF136, ZNF137, ZNF138, ZNF14, ZNF140, ZNF141, ZNF146, ZNF155, ZNF157, ZNF160, ZNF169, ZNF174, ZNF175, ZNF177, ZNF180, ZNF182, ZNF185, ZNF189, ZNF19, ZNF20, ZNF200, ZNF205, ZNF208, ZNF212, ZNF213, ZNF217, ZNF219, ZNF22, ZNF221, ZNF222, ZNF223, ZNF224, ZNF225, ZNF226, ZNF229, ZNF23, ZNF230, ZNF234, ZNF235, ZNF239, ZNF254, ZNF26, ZNF263, ZNF266, ZNF267, ZNF268, ZNF273, ZNF275, ZNF277, ZNF28, ZNF282, ZNF295, ZNF3, ZNF30, ZNF319, ZNF32, ZNF331, ZNF334, ZNF337, ZNF33B, ZNF354A, ZNF358, ZNF37A, ZNF41, ZNF410, ZNF414, ZNF415, ZNF423, ZNF43, ZNF430, ZNF432, ZNF44, ZNF443, ZNF45, ZNF461, ZNF473, ZNF483, ZNF510, ZNF559, ZNF576, ZNF613, ZNF629, ZNF630, ZNF646, ZNF649, ZNF668, ZNF682, ZNF701, ZNF706, ZNF708, ZNF711, ZNF746, ZNF75A, ZNF79, ZNF81, ZNF823, ZNF83, ZNF831, ZNF84, ZNF85, ZNF90, ZNF91, ZNF92, ZNF93, ZSCAN10, ZSCAN21, ZXDA, ZXDB | AATK, ABL1, ACVR1B, ACVRL1, ADCK1, ADCK2, **AKT1, AKT2**, ALPK1, AMHR2, ARAF, AURKA, AXL, BCKDK, BLK, BMP2K, BMPR1B, BMX, BRAF, BRD3, BRD4, BTK, C9orf96, CAMK1D, CAMK2A, CAMK2B, CAMK2D, CAMK2G, CAMK4, CAMKK2, CASK, CDC42BPB, CDK10, CDK13, CDK14, CDK16, CDK17, CDK2, CDK20, CDK3, CDK4, CDK5, CDK6, CDK7, CDK9, CDKL1, CDKL2, CDKL3, CDKL5, CHUK, CIT, CLK4, CSF1R, CSNK1A1, **CSNK1D**, **CSNK1G2**, CSNK1G3, CSNK2A1, CSNK2A2, DAPK1, DAPK3, DCLK2, DMPK, DYRK1A, DYRK1B, DYRK2, DYRK4, EEF2K, EGFR, EIF2AK1, **EPHA1, EPHA5, EPHA6, EPHB4, EPHB6,** ERBB3, ERN1, ERN2, FASTK, FER, FGFR1, FGFR2, FGFR3, FGFR4, FLT4, GAK, GRK4, GRK5, GRK6, GSK3A, GUCY2C, GUCY2F, HCK, HIPK2, HIPK4, HSPB8, HUNK, IKBKB, INSR, IRAK1, IRAK3, IRAK4, ITK, JAK2, JAK3, KDR, KIT, KSR2, LIMK1, LMTK2, LMTK3, LRRK2, LYN, MAP2K2, MAP2K6, MAP2K7, MAP3K1, MAP3K10, MAP3K12, MAP3K15, MAP3K3, MAP3K9, MAP4K1, MAP4K5, MAPK10, MAPK3, MAPK9, MAPKAPK5, MARK3, MARK4, MAST1, MAST3, MAST4, MATK, MELK, MET, MGC42105, MKNK2, MLKL, MOS, MST4, MUSK, MYLK2, MYLK3, MYO3A, NEK1, NEK4, NEK6, NEK9, NPR2, NRK, NTRK2, NUAK1, NUMBL, PAK3, PAK4, PAK7, PDGFRA, PDGFRB, PDK3, PDK4, PDPK1, PHKG1, PHKG2, PIM2, PKMYT1, PKN1, PKN3, PLK1, PLK2, PLK4, PNCK, PRKAA1, **PRKACA**, PRKACG, PRKCA, PRKCB, PRKCG, PRKCH, PRKCQ, PRKD1, PRKD2, PRKDC, PRKG1, PRKG2, PRKX, PRKY, PSKH1, PSKH2, PTK2, PTK6, RAGE, **RET**, RIOK2, RIPK2, RIPK3, RIPK4, ROR2, RPS6KA3, RPS6KA5, RPS6KA6, RPS6KL1, SBK1, SCYL2, SGK196, **SGK2, SGK3**, SIK1, SLK, SMG1, SRC, SRMS, SRPK2, STK10, STK11, STK17A, STK3, STK31, STK32A, STK32B, STK32C, STK35, STK38L, STK4, STRADA, STYK1, SYK, TAF1, TAF1L, TAOK2, TAOK3, TBCK, TBK1, TEC, TEK, TESK1, TGFBR1, TLK2, TP53RK, TRIB1, TRIB3, TRIM24, TRIO, TRPM6, TRRAP, TSSK1B, TSSK4, TSSK6, TWF1, TXK, TYK2, ULK1, VRK1, VRK3, WEE2, WNK1, WNK2, WNK3 | ABL1, ACSL6, AFF1, AFF4, AKAP9, **AKT1, AKT2,** ARHGAP26, ASPSCR1, ATF1, BCL11B, BCL3, BCL7A, BRAF, BRD3, BRD4, BTG1, CANT1, CARD11, CBFA2T3, CBFB, CCNB1IP1, CCND2, CD74, CD79A, CD79B, CDH11, CDK4, CDK6, CEBPA, CEP110, CHCHD7, CHIC2, CIC, CIITA, CLTC, COX6C, CREB3L2, CREBBP, CRLF2, CRTC1, DDIT3, DDX5, DUX4, EGFR, ELF4, ELL, ELN, ERC1, ERG, ETV1, ETV6, FGFR1, FGFR2, FGFR3, FIP1L1, FNBP1, FOXO4, FSTL3, FUS, GATA1, GNAQ, **GNAS**, GOLGA5, GPHN, HERPUD1, HIP1, HMGA2, HNRNPA2B1, HOOK3, HOXA11, HOXA13, HOXA9, HOXC11, HOXC13, HSP90AA1, IKZF1, IL2, IL21R, IL6ST, ITK, JAK2, JAK3, JAZF1, KDM5A, KDR, KIAA1549, KIT, KLK2, KRAS, KTN1, LIFR, LPP, LYL1, MAF, MAFB, MDM2, MET, MLLT1, MLLT3, MNX1, MSN, MTCP1, MYC, MYH11, MYST3, MYST4, NACA, NCOA2, NFIB, NFKB2, NIN, NONO, **NOTCH1**, NPM1, NR4A3, NSD1, NUP214, OLIG2, OMD, P2RY8, PAX5, PCM1, PDGFRA, PDGFRB, PLAG1, PSIP1, PTPN11, RAD51L1, RANBP17, RAP1GDS1, **RET**, RHOH, RNF213, RUNX1, RUNX1T1, SET, SH3GL1, SMO, SS18L1, SSX1, SSX2, SSX4, SYK, TAL2, TCEA1, TCF3, TCL1A, TCL6, TET1, TFE3, TLX1, TLX3, TMPRSS2, TNFRSF17, TOP1, TPM4, TRIM24, TRIP11, TSHR, WHSC1, WHSC1L1, ZNF331, ZNF384 | APC, ASXL1, BRIP1, CBLC, CDH1, CDKN2A, CYLD, DICER1, **ERCC2, ERCC4,** EXT1, FAM123B, FANCA, FANCC, FANCG, FAS, **FBXW7**, GATA3, HNF1A, KDM5C, KDM6A, KLF6, NBN, PALB2, PHOX2B, PIK3R1, PMS2, PRF1, PTCH1, **PTEN**, SBDS, SMARCA4, SOCS1, STK11, SUFU, TET2, **TSC1, TSC2**, WRN, XPA |
| **Carcinoma – Frequent CN losses (n=4/22)** | | | | |
| ADM2, AGT, ANGPTL7, ARTN, BMP8A, BMP8B, CCL1, CCL11, CCL14, CCL14-CCL15, CCL15, CCL16, CCL18, CCL2, CCL23, CCL3, CCL3L1, CCL3L3, CCL4, CCL4L1, CCL4L2, CCL5, CCL7, CCL8, CLCF1, CORT, CSF3, CSH1, CSH2, CSHL1, CXCL16, EDN2, FGF11, FGF14, FGF9, GAL, GAST, GH1, GH2, GIP, GPHA2, GRN, GUCA2A, **IGF2**, IL17D, **INS, INS-IGF2,** INSL5, KL, LEFTY1, LEFTY2, LIF, LTBP3, MIF, NPPA, NPPB, NPPC, OSM, PDGFB, PPY, PRLH, RABEP1, SCT, SECTM1, TNFSF11, TNFSF12, TNFSF13, TNFSF13B, TXLNA, TYMP, UTS2 | AATF, ARID3B, ASCL2, ATF4, ATG4B, BARHL2, BARX2, BPTF, BRD1, BRDT, BTF3L1, C13orf15, C22orf31, CASZ1, CBX1, CBX2, CBX4, CBX6, CBX7, CBX8, CDK8, CDX2, CHD3, CHD5, CREBBP, DACH1, DEAF1, DLX3, DLX4, DMBX1, DMRT1, DMRTA2, DPF2, DR1, DRAP1, E2F2, ELF1, ELF4, ENO1, EP300, ETS1, ETV4, EYA3, EZH1, FAM48A, FLI1, FOSL1, FOXD2, FOXD3, FOXE3, FOXJ1, FOXJ3, FOXK2, FOXN1, FOXO1, FUBP1, GATA1, GATA6, GFI1, GLIS1, GSC2, GTF2F2, GTF3A, GTPBP1, HDAC1, HDAC4, **HES2, HES4, HES5, HES6, HES7,** HEXIM1, HEYL, HIC1, HIC2, HIRA, HLF, HMGB1, HMGN2, HNF1B, HOXB1, HOXB13, HOXB2, HOXB3, HOXB4, HOXB5, HOXB6, HOXB7, HOXB8, HOXB9, HSFY1, ID3, IKZF3, ILF2, INTS4, IRF2, IRF7, JMJD4, **JUN,** KAT2A, KAT5, KDM4C, KLF12, KLF5, KLHL21, LHX1, LMO7, LZTR1, MAFF, MAFG, MBD1, MBNL2, MED1, MED15, MED24, MEIS3P1, MEOX1, MICALL1, MIER1, MIXL1, MLLT6, MLX, MNT, MSX2P1, MTF1, MTF2, MYBBP1A, MYCBP, MYCL1, MYST2, NCOA1, NCOR1, NEUROD2, NFATC1, NFE2L1, NFIA, NFRKB, NFX1, NFYC, NR0B2, NR1D1, NR5A2, NUFIP2, OVOL1, PATZ1, PAX7, PCGF2, PDX1, PER1, PER2, PER3, PHF11, PHRF1, PIAS2, PKNOX2, POU2F3, POU3F1, POU4F1, PPARA, PPARD, PRDM10, PRDM16, PRDM2, PSMC5, RARA, RASSF7, RB1, RCVRN, RELA, RERE, RIMS3, RLF, RNF112, RORC, RUNX3, SALL3, SAP18, SAP30BP, SETBP1, SF1, SFRS17A, SHOX, SMAD2, SMAD3, SMAD4, SMAD6, SMAD7, SMAD9, SMARCAL1, SMARCB1, SMARCE1, SMCR7L, SNAPC5, SOX1, SOX10, SOX13, SOX15, SOX21, SOX9, SP2, SPEN, SREBF1, SREBF2, SRY, STAT3, STAT5A, STAT5B, SUPT4H1, SUPT6H, SUV420H1, TADA2A, TAF12, TAF13, TAF15, TAF4B, TAF5L, TAF6L, TAL1, TBL1Y, TBX1, TBX10, TBX2, TBX21, TBX4, TCF20, TEAD3, TEF, TFB1M, TFDP1, TGIF1, THOC4, THRA, THRAP3, **TP53**, TP53I13, **TP73**, TRIM13, TRIM25, TRIM62, TRIOBP, TSC22D1, TSHZ1, TTLL4, TULP1, UBR4, UBTF, ULK2, VEZF1, WDR45L, XBP1, YBX1, YBX2, ZBTB17, ZBTB40, ZBTB48, ZFP161, ZFPL1, ZFY, ZIC2, ZIC5, ZMYM4, ZMYM6, ZNF142, ZNF143, ZNF18, ZNF195, ZNF202, ZNF207, ZNF232, ZNF236, ZNF24, ZNF271, ZNF281, ZNF286A, ZNF287, ZNF362, ZNF516, ZNF593, ZNF643, ZNF672, ZNF691, ZNF692, ZNF74, ZSCAN20 | AATK, ADRBK1, ADRBK2, ALPK2, ATM, AURKB, BCR, BRDT, BRSK2, CABC1, CAMK2G, CAMKK1, CDC42BPA, CDC42BPG, CDC7, CDK11A, CDK12, CDK3, CDK8, CHEK1, CHEK2, CLK3, CLUL1, CSNK1A1L, **CSNK1D**, CSNK1E, **EPHA10, EPHA2, EPHA8, EPHB2,** ERBB2, ERN1, FGR, FLT1, FLT3, GRK1, GSG2, GUCY2D, JAK1, KIAA1804, KSR1, LATS1, LATS2, LCK, LIMK2, MAP2K1, MAP2K3, MAP2K4, MAP2K5, MAP2K6, MAP3K11, MAP3K14, MAP3K3, MAP3K6, MAP4K2, MAPK1, MAPK11, MAPK12, MAPK4, MAPK7, MARK2, MAST2, MELK, MINK1, MKNK1, **MTOR**, NEK3, NEK5, NEK8, NLK, NPR1, OBSCN, PASK, PDIK1L, PDK2, PIM3, PINK1, PLK3, PRKAA2, PRKCA, PRKCB, PRKCZ, PRKY, RIOK3, ROCK1, ROR1, RPS6KA1, RPS6KB2, SCYL1, **SGK1,** SGK269, STK16, STK24, STK25, STK36, STK40, STRADA, TAOK1, TESK2, TEX14, TIE1, TLK2, TNK1, TSSK2, TSSK3, ULK2, WEE1 | ARHGEF12, ASPSCR1, BCL2, BCR, CANT1, CARS, CD79B, CDX2, CLTC, CLTCL1, COL1A1, CREBBP, CRLF2, CYTSB, DDX10, DDX5, ELF4, EPS15, ERBB2, ETV4, EWSR1, FEV, FLI1, FLT3, FOXO1, GAS7, GATA1, HLF, **HRAS,** JAK1, JUN, KDSR, LASP1, LCK, LCP1, LHFP, LPP, MDS2, MKL1, MLLT6, MN1, MPL, MYCL1, MYH9, NCOA1, NONO, NUP98, P2RY8, PATZ1, PAX7, PDGFB, PER1, PRDM16, RABEP1, RARA, RNF213, RPL22, SEPT5, SEPT9, SFPQ, SS18, STIL, SUZ12, TAF15, TAL1, THRAP3, ZMYM2, ZNF52 | ATM, BRCA1, BRCA2, BRIP1, CDKN2A, CDKN2C, CHEK2, EP300, E**RCC5**, MAP2K4, MEN1, MUTYH, NF1, NF2, RB1, SDHAF2, SDHB, SMAD4, SMARCB1, **TP53** |
| **Carcinoma – Frequent cnLOH (n=4/22)** | | | | |
| ADIPOQ, ADM, ADM2, AGT, AMBN, AMELX, ANGPTL5, ANGPTL7, APLN, AREG, ARTN, BDNF, BMP1, BMP10, BMP15, BMP3, BMP5, BMP6, BMP8A, BMP8B, BTC, C5, CALCA, CALCB, CAMP, CAT, CCK, CCL1, CCL11, CCL13, CCL14, CCL14-CCL15, CCL15, CCL16, CCL18, CCL19, CCL2, CCL20, CCL21, CCL23, CCL27, CCL3, CCL3L1, CCL3L3, CCL4, CCL4L1, CCL4L2, CCL5, CCL7, CCL8, CD40LG, CER1, CGA, CHGA, CKLF, CLCF1, CMTM1, CMTM2, CMTM4, CMTM6, CMTM7, CMTM8, CNTF, CORT, CRH, CSF1, CSF3, CSH1, CSH2, CSHL1, CSPG5, CTGF, CXCL1, CXCL10, CXCL11, CXCL12, CXCL13, CXCL16, CXCL2, CXCL3, CXCL5, CXCL6, CXCL9, CYR61, DEFA1, DEFA3, DEFA5, DEFB1, DEFB103A, DEFB104A, DEFB4, DKK1, EDN1, EDN2, EGF, EPGN, EREG, FAM3B, FAM3D, FASLG, FGF11, FGF12, FGF13, FGF14, FGF16, FGF17, FGF19, FGF2, FGF20, FGF3, FGF4, FGF5, FGF7, FGF8, FGF9, FIGF, FSHB, GAL, GAST, GCG, GDF10, GDF2, GDF6, GDF7, GH1, GH2, GHRL, GIP, GKN1, GNRH1, GPHA2, GREM1, GREM2, GRN, GRP, GUCA2A, HDGF, HDGFRP3, HTN3, IFNA1, IFNA10, IFNA13, IFNA14, IFNA16, IFNA17, IFNA2, IFNA21, IFNA4, IFNA5, IFNA6, IFNA7, IFNA8, IFNB1, IFNE, IFNK, IFNW1, **IGF2,** IL10, IL12A, IL15, IL16, IL17A, IL17D, IL17F, IL18, IL19, IL1A, IL1B, IL1F10, IL1F5, IL1F6, IL1F7, IL1F8, IL1F9, IL1RN, IL2, IL20, IL21, IL24, IL32, IL33, IL7, IL8, INHA, INHBB, **INS, INS-IGF2,** INSL4, INSL5, INSL6, KGFLP, KGFLP2, KL, LEFTY1, LEFTY2, LIF, LRSAM1, LTA, LTB, LTBP1, LTBP3, MDK, MIF, MLN, MSTN, NDP, NENF, NGF, NMB, NODAL, NOV, NPPA, NPPB, NPPC, NRG1, NRG3, NRG4, NUDT6, OGN, OSGIN1, OSM, OSTN, PDGFB, PDGFC, PDGFD, PDGFRA, PDGFRL, PENK, PF4, PF4V1, PLAU, PNOC, **POMC,** PPBP, PPBPL1, PPBPL2, PPY, PRL, PRLH, PROK1, PROK2, PTH, PYY, QRFP, RABEP1, REG1A, RETNLB, RLN1, RLN2, S100A6, SAA1, SAA2, SCG2, SCT, SECTM1, SEMA3B, SEMA3F, SEMA3G, SEMA4A, SEMA4B, SEMA4C, SEMA4D, SEMA4F, SEMA4G, SEMA5B, SEMA6C, SEMA6D, SEMA7A, SLIT1, SLIT2, SLURP1, SPP1, SST, STC1, TDGF1, TDGF3, TG, **TGFA, TGFB2**, THPO, TNC, TNF, TNFRSF11B, TNFSF10, TNFSF11, TNFSF12, TNFSF13, TNFSF13B, TNFSF15, TNFSF18, TNFSF4, TNFSF8, TOR2A, TRH, TSHB, TXLNA, TYMP, UCN, UCN2, UCN3, UTS2, UTS2D, **VEGFA, VEGFB, VEGFC,** VIP, XCL1, XCL2 | AATF, ABT1, ACVR2A, ADPGK, AFF1, AFF2, AFF3, **AIRE**, ALX3, ALX4, ANP32A, **AR**, ARC, ARFGAP2, ARID1B, ARID3B, ARID5A, ARID5B, ARIH2, ARNT, ARNT2, ARNTL, ASCL2, ASCL3, ASH1L, ASH2L, ATF2, ATF3, ATF4, ATF6, ATF6B, ATG4B, ATOH1, ATOH7, ATRX, BACH1, BACH2, BANP, BARHL1, BARHL2, BARX1, BARX2, BATF3, BAZ2B, BCL11A, BCL6, BCLAF1, BHLHE22, BHLHE40, BLZF1, BMI1, BNC1, BNC2, BPTF, BRD1, BRD2, BRD3, BRDT, BRF1, BRPF1, BRPF3, BTAF1, BTF3L1, C11orf9, C13orf15, C15orf42, C1orf61, C22orf31, CASK, CASZ1, CBX1, CBX2, CBX4, CBX6, CBX7, CBX8, CCT4, CDK8, CDX2, CDX4, CEBPD, CEBPZ, CHD1L, CHD2, CHD3, CHD5, CIAO1, CIR1, CITED1, CITED2, CLOCK, CNBP, CNPY3, COPS2, COPS5, CREB1, CREB3, CREB3L1, CREBZF, CREG1, CREM, CRIP1, CRIP2, CSRP1, CSRP3, **CTNNB1**, DACH1, DCP1A, DEAF1, DENND4A, DEPDC7, DLX1, DLX2, DLX3, DLX4, DMBX1, DMRT1, DMRT2, DMRTA2, DMRTC1, DPF2, DPF3, DR1, DRAP1, DUX4, E2F2, E2F3, E2F5, E2F6, E4F1, ECD, EED, EGR2, EGR3, EGR4, EHF, ELF1, ELF2, ELF3, ELF4, ELF5, ELK1, ELK4, EMX1, EMX2, EN1, ENO1, EOMES, EP300, EPAS1, **ERCC3, ERCC6**, ERG, ERMP1, ESR1, ESRRA, ESRRB, ESRRG, ETS1, ETS2, ETV3, ETV4, ETV5, ETV7, EVX2, EYA1, EYA3, EYA4, EZH1, FAM189B, FAM48A, FEZF2, FHL1, FHL2, FHL5, FLI1, FOS, FOSL1, FOSL2, FOXB1, FOXC1, FOXD2, FOXD3, FOXD4, FOXE1, FOXE3, FOXF2, FOXH1, FOXJ1, FOXJ3, FOXK2, FOXL2, FOXN1, FOXN2, FOXN3, FOXO1, FOXO3, FOXO4, FOXP1, FOXP3, FOXQ1, FUBP1, GABPA, GABPB1, GATA1, GATA2, GATA3, GATA4, GATA6, GBX2, GCM1, GCM2, GFI1, GFI1B, GLI2, GLIS1, GLIS2, GRHL1, GSC, GSC2, GSX2, GTF2A1, GTF2A1L, GTF2A2, GTF2B, GTF2E1, GTF2E2, GTF2F2, GTF2H1, GTF2H4, GTF3A, GTF3C2, GTF3C3, GTF3C4, GTF3C5, GTPBP1, HAND2, HDAC1, HDAC2, HDAC4, **HES1, HES2, HES4, HES5, HES6, HES7,** HESX1, HEXIM1, HEY1, HEY2, HEYL, HHEX, HIC1, HIC2, HIF1A, HIRA, HIVEP1, HIVEP2, HLF, HLTF, HLX, HMGA1, HMGB1, HMGB2, HMGN2, HMX2, HMX3, HNF1B, HNF4G, HOPX, HOXB1, HOXB13, HOXB2, HOXB3, HOXB4, HOXB5, HOXB6, HOXB7, HOXB8, HOXB9, HOXD1, HOXD10, HOXD11, HOXD12, HOXD13, HOXD3, HOXD4, HOXD8, HOXD9, HR, HSF1, HSF2, HSF2BP, HSFX1, HSFY1, ID2, ID3, ID4, IFT172, IGHMBP2, IKZF2, IKZF3, IKZF5, ILF2, INTS4, IRF2, IRF4, IRF6, IRF7, ISL2, JARID2, JMJD4, **JUN,** KAT2A, KAT5, KCNIP3, KDM4C, KDM4D, KDM5C, KIAA0040, KLF10, KLF11, KLF12, KLF13, KLF15, KLF3, KLF4, KLF5, KLF6, KLF7, KLF8, KLF9, KLHL21, KLHL5, LAS1L, LBX1, LDB1, LDB2, LDOC1, **LEF1,** LHX1, LHX2, LHX3, LHX4, LHX6, LHX8, LHX9, LMO1, LMO2, LMO4, LMO7, LMX1A, LMX1B, LOC151162, LRRC14, LZTR1, LZTS1, MAFF, MAFG, MAMLD1, MAPK8IP1, MBD1, MBD2, MBD4, MBNL2, MECOM, MECP2, MED1, MED12, MED14, MED15, MED17, MED23, MED24, MED27, MED6, MEF2A, MEF2D, MEFV, MEIS1, MEIS2, MEIS3P1, MEOX1, MESP2, MFSD3, MGA, MICALL1, MID1, MIER1, MITF, MIXL1, MLL, MLLT10, MLLT3, MLLT4, MLLT6, MLX, MNDA, MNT, MORF4, MORF4L1, MORF4L2, MSC, MSRB2, MSX1, MSX2P1, MTA1, MTA2, MTF1, MTF2, MXD1, MXD4, MXI1, MYB, MYBBP1A, MYBL1, **MYC**, MYCBP, MYCL1, MYCN, MYCNOS, MYOD1, MYOG, MYST2, MYST3, MYST4, MYT1L, NAB1, NCALD, NCOA1, NCOA2, NCOA4, NCOR1, NEUROD1, NEUROD2, NEUROG2, NEUROG3, NFAT5, NFATC1, NFATC3, NFE2L1, NFE2L2, NFIA, NFIB, NFIL3, **NFKB1,** NFKB2, NFKBIE, NFKBIL1, NFKBIL2, NFRKB, NFX1, NFYA, NFYC, NHLH2, NKRF, NKX2-3, NKX3-1, NKX3-2, NKX6-1, NKX6-2, NMI, NPAS2, NR0B1, NR0B2, NR1D1, NR1D2, NR1H3, NR1I2, NR1I3, NR2C2, NR2E1, NR2E3, NR2F2, NR3C2, NR4A2, NR4A3, **NR5A1,** NR5A2, NR6A1, NRIP1, NUFIP2, OLIG1, OLIG2, OLIG3, ONECUT1, ONECUT2, OPTN, OSR1, OTUD7B, OTX1, OVOL1, PACS2, PATZ1, PAX2, PAX3, PAX5, PAX6, PAX7, PAX8, PBX1, PBX2, PBX3, PCGF2, PCGF3, PDLIM5, PDX1, PER1, PER2, PER3, PGR, PHF11, PHF3, PHOX2A, PHOX2B, PHRF1, PHTF1, PIAS2, PIAS3, PITX2, PITX3, PKNOX1, PKNOX2, PLAG1, PLAGL1, PLRG1, PMF1, PML, **POU1F1,** POU2AF1, POU2F1, POU2F3, POU3F1, POU3F2, POU3F3, POU3F4, POU4F1, POU4F2, POU5F1, PPARA, PPARD, PPARG, PPARGC1A, PRDM1, PRDM10, PRDM11, PRDM12, PRDM13, PRDM14, PRDM15, PRDM16, PRDM2, PRDM5, PRDM8, PREB, PRICKLE3, PROX1, PRRX1, PRRX2, PSIP1, PSMC5, PTTG1IP, RARA, RARB, RASSF7, RAX, RB1, RBBP5, RBBP9, RBPJ, RCVRN, REL, RELA, RERE, REST, REV3L, RFX3, RFX5, RIMS3, RING1, RLF, RLIM, RNF103, RNF112, RNF113A, RNF115, RNF13, RNF141, RNF144A, RNF160, RNF2, RNF4, RNF8, RORA, RORB, RORC, RREB1, RUNX1, RUNX1T1, RUNX2, RUNX3, RXRA, RXRB, RXRG, SALL3, SAP18, SAP30, SAP30BP, SATB1, SCAND2, SCML1, SCML2, SCRT1, SERTAD2, SETBP1, SETD4, SETDB1, SF1, SFRS17A, SHOX, SHOX2, SIAH2, SIM1, SIM2, SIX2, SIX3, SMAD1, SMAD2, SMAD3, SMAD4, SMAD6, SMAD7, SMAD9, SMARCA1, SMARCA2, SMARCA5, SMARCAD1, SMARCAL1, SMARCB1, SMARCC1, SMARCE1, SMCR7L, SMYD5, SNAI2, SNAPC1, SNAPC3, SNAPC4, SNAPC5, SNW1, SOX1, SOX10, SOX11, SOX13, SOX14, SOX15, SOX17, SOX2, SOX21, SOX3, SOX4, SOX6, SOX9, SP2, SP3, SPDEF, SPEN, SPI1, SREBF1, SREBF2, SRF, SSRP1, SSX1, SSX2, SSX3, SSX4, SSX5, SSX6, SSX7, SSX8, SSX9, ST18, STAT1, STAT3, STAT4, STAT5A, STAT5B, STON1, SUPT3H, SUPT4H1, SUPT6H, SUV420H1, TADA2A, TADA3, TAF1, TAF10, TAF11, TAF12, TAF13, TAF15, TAF1A, TAF1B, TAF1C, TAF2, TAF3, TAF4B, TAF5, TAF5L, TAF6L, TAL1, TAL2, TBL1Y, TBP, TBPL1, TBR1, TBX1, TBX10, TBX15, TBX18, TBX19, TBX2, TBX21, TBX22, TBX4, TCEAL1, TCF12, TCF19, TCF20, TCF21, TCF4, TCF7L1, TCF7L2, TEAD1, TEAD3, TEF, TFAM, TFAP2A, TFAP2B, TFAP2D, TFAP4, TFB1M, TFB2M, TFCP2L1, TFDP1, TFDP2, TFDP3, TFE3, TFEB, TGIF1, THOC4, THRA, THRAP3, THRB, TLX1, TLX2, TMF1, TOX, **TP53,** TP53I13, TP63, **TP73,** TRIM10, TRIM13, TRIM15, TRIM22, TRIM25, TRIM26, TRIM27, TRIM3, TRIM33, TRIM38, TRIM62, TRIOBP, TRIP11, TRIP4, TRPS1, TSC22D1, TSC22D2, TSC22D3, TSHZ1, TTLL4, TTLL5, TUB, TULP1, TULP4, UBP1, UBR4, UBTF, ULK2, USF1, UTF1, VAX1, VAX2, VENTX, VEZF1, VGLL1, VPS24, VPS72, WBP5, WDR45L, WHSC1, WT1, XBP1, YBX1, YBX2, ZAR1, ZBTB11, ZBTB16, ZBTB17, ZBTB20, ZBTB22, ZBTB24, ZBTB33, ZBTB38, ZBTB40, ZBTB43, ZBTB47, ZBTB48, ZBTB6, ZBTB7B, ZEB1, ZEB2, ZFAND3, ZFAND5, ZFAND6, ZFP106, ZFP161, ZFP36L2, ZFP37, ZFP91, ZFP92, ZFPL1, ZFPM2, ZFX, ZHX1, ZIC1, ZIC2, ZIC3, ZIC4, ZIC5, ZKSCAN3, ZKSCAN4, ZMYM3, ZMYM4, ZMYM5, ZMYM6, ZMYND11, ZNF124, ZNF133, ZNF141, ZNF142, ZNF143, ZNF148, ZNF157, ZNF16, ZNF165, ZNF167, ZNF169, ZNF174, ZNF18, ZNF182, ZNF184, ZNF185, ZNF187, ZNF189, ZNF192, ZNF193, ZNF195, ZNF197, ZNF2, ZNF200, ZNF202, ZNF205, ZNF207, ZNF213, ZNF214, ZNF215, ZNF22, ZNF232, ZNF236, ZNF238, ZNF239, ZNF24, ZNF248, ZNF25, ZNF250, ZNF251, ZNF259, ZNF263, ZNF271, ZNF275, ZNF281, ZNF286A, ZNF287, ZNF295, ZNF318, ZNF32, ZNF33A, ZNF33B, ZNF35, ZNF362, ZNF37A, ZNF395, ZNF408, ZNF41, ZNF483, ZNF510, ZNF516, ZNF532, ZNF592, ZNF593, ZNF630, ZNF639, ZNF643, ZNF669, ZNF672, ZNF691, ZNF692, ZNF696, ZNF7, ZNF706, ZNF711, ZNF74, ZNF75A, ZNF76, ZNF79, ZNF80, ZNF81, ZNHIT6, ZRANB2, ZSCAN10, ZSCAN12, ZSCAN2, ZSCAN20, ZXDA, ZXDB, ZXDC | AAK1, AATK, ABL1, ABL2, ACVR1, ACVR1C, ACVR2A, ACVR2B, ADCK1, ADCK5, ADRBK1, ADRBK2, AKT3, ALK, ALPK1, ALPK2, ALPK3, ANKK1, ARAF, ATM, ATR, AURKB, BCR, BLK, BMP2K, BMPR1A, BMPR1B, BMPR2, BMX, BRD2, BRD3, BRDT, BRSK2, BTK, BUB1, BUB1B, C9orf96, CABC1, CAMK1, CAMK1D, CAMK1G, CAMK2D, CAMK2G, CAMKK1, CAMKV, CASK, CDC42BPA, CDC42BPG, CDC7, CDK1, CDK11A, CDK12, CDK15, CDK16, CDK18, CDK19, CDK20, CDK3, CDK8, CDK9, CDKL2, CDKL4, CDKL5, CHEK1, CHEK2, CHUK, CLK1, CLK2, CLK3, CLUL1, CSK, CSNK1A1L, CSNK1D, CSNK1E, CSNK1G1, DAPK1, DAPK2, DCLK2, DCLK3, DDR1, DDR2, DSTYK, DYRK1A, DYRK3, EIF2AK2, EIF2AK3, EIF2AK4, **EPHA10, EPHA2, EPHA3, EPHA4, EPHA5, EPHA6, EPHA7, EPHA8, EPHB1, EPHB2, EPHB3,** ERBB2, ERBB4, ERN1, FES, FGFR1, FGFR2, FGFR3, FGR, FLT1, FLT3, FRK, FYN, GAK, GRK1, GRK4, GRK5, GRK7, GSG2, GSK3B, GUCY2D, GUCY2F, HIPK1, HIPK3, HUNK, ICK, **IGF1R,** IKBKB, IKBKE, ILK, INSRR, IRAK1, IRAK2, JAK1, JAK2, KALRN, KDR, KIAA1804, KIT, KSR1, LATS1, LATS2, LCK, LIMK2, LRRK1, LTK, LYN, MAK, MAP2K1, MAP2K3, MAP2K4, MAP2K5, MAP2K6, MAP3K11, MAP3K13, MAP3K14, MAP3K15, MAP3K2, MAP3K3, MAP3K4, MAP3K5, MAP3K6, MAP3K7, MAP3K8, MAP3K9, MAP4K2, MAP4K3, MAP4K4, MAPK1, MAPK10, MAPK11, MAPK12, MAPK13, MAPK14, MAPK15, MAPK4, MAPK6, MAPK7, MAPK8, MAPKAPK2, MAPKAPK3, MARK1, MARK2, MAST2, MASTL, MELK, MERTK, MINK1, MKNK1, MOS, MST1R, MST4, MTOR, MUSK, MYLK, MYLK4, MYO3A, MYO3B, NEK1, NEK10, NEK11, NEK2, NEK3, NEK4, NEK5, NEK6, NEK7, NEK8, NLK, NPR1, NPR2, NRBP1, NRBP2, NRK, NTRK1, NTRK2, NTRK3, NUAK2, OBSCN, OXSR1, PAK1, PAK3, PAK6, PASK, PBK, PDGFRA, PDIK1L, PDK1, PDK2, PDK3, PDPK1, PIK3R4, PIM1, PIM2, PIM3, PINK1, PKDCC, PKMYT1, PKN2, PKN3, PLK3, PLK4, PNCK, PRKAA2, PRKACB, PRKACG, PRKCA, PRKCD, PRKCE, PRKCH, PRKCI, PRKCQ, PRKCZ, PRKD1, PRKD3, PRKDC, PRKG1, PRKG2, PRKX, PRKY, PRPF4B, PSKH1, PSKH2, PTK2, PTK2B, PTK7, PXK, RAF1, RAGE, **RET**, RIOK1, RIOK3, RIPK1, RIPK2, RIPK4, RNASEL, ROCK1, ROCK2, ROR1, ROR2, ROS1, RPS6KA1, RPS6KA2, RPS6KA3, RPS6KA4, RPS6KA5, RPS6KA6, RPS6KB2, RPS6KC1, RYK, SCYL1, SCYL3, **SGK1**, SGK196, SGK269, **SGK3**, SIK1, SIK2, SIK3, SLK, SNRK, SPEG, SRPK1, STK16, STK17B, STK19, STK24, STK25, STK3, STK32B, STK32C, STK33, STK36, STK38, STK39, STK40, STRADA, STRADB, SYK, TAF1, TAF1L, TAOK1, TBCK, TEC, TEK, TESK1, TESK2, TEX14, **TGFBR1, TGFBR2**, TIE1, TLK1, TLK2, TNIK, TNK1, TNK2, TNNI3K, TRIB1, TRIB2, TRIM33, TRPM6, TRPM7, TSSK2, TSSK3, TTBK1, TTBK2, TTK, TTN, TWF2, TXK, TYRO3, UHMK1, ULK2, ULK3, ULK4, VRK1, VRK2, WEE1, WNK2, WNK3, WNK4, YSK4, ZAK, ZAP70 | ABI1, ABL1, ABL2, ACSL3, AFF1, AFF3, ALK, ARHGEF12, ARNT, ASPSCR1, ATIC, BCL10, BCL11A, BCL2, BCL6, BCL9, BCR, BIRC3, BRD3, C15orf21, C15orf55, CANT1, CARS, CASC5, CCDC6, CCND1, CCND3, CD79B, CDX2, CEP110, CHCHD7, CHIC2, CHN1, CLP1, CLTC, CLTCL1, CNBP, COL1A1, COX6C, CREB1, CRLF2, CRTC3, **CTNNB1,** CXCR7, CYTSB, DDX10, DDX5, DDX6, DEK, DUX4, EIF4A2, ELK4, EML4, EPS15, ERBB2, ETV4, ETV5, EWSR1, FCGR2B, FCRL4, FEV, FGFR1,FGFR1OP, FGFR2, FGFR3, FIP1L1, FLI1, FLT3, FNBP1, FOXL2, FOXO1, FOXO3, FOXO4, FOXP1, GAS7, GATA2, GMPS, GNAQ, GOLGA5, GOPC, HIST1H4I, HLF, HMGA1, HOOK3, HOXD11, HOXD13, **HRAS,** HSP90AA1, HSP90AB1, IDH1, IDH2, IL2, IRF4, JAK1, JAK2, **JUN**, KDR, KDSR, KIT, LASP1, LCK, LCP1, LHFP, LMO1, LMO2, LPP, MALT1, MAML2, MDM4, MDS2, MECOM, MITF, MKL1, MLF1, MLL, MLLT10, MLLT11, MLLT3, MLLT4, MLLT6, MN1, MPL, MSI2, MSN, MUC1, MYB, **MYC**, MYCL1, MYCN, MYH9, MYST3, MYST4, NCKIPSD, NCOA1, NCOA2, NCOA4, NFIB, NFKB2, **NOTCH1, NOTCH2,** NR4A3, NRAS, NTRK1, NTRK3, NUMA1, NUP214, NUP98, OMD, P2RY8, PAFAH1B2, PATZ1, PAX3, PAX5, PAX7, PAX8, PBX1, PCM1, PCSK7, PDE4DIP, PDGFB, PDGFRA, PER1, PICALM, PIK3CA, PIM1, PLAG1, PML, POU2AF1, POU5F1, PPARG, PRCC, PRDM16, PRRX1, PSIP1, RABEP1, RAF1, RAP1GDS1, RARA, RBM15, REL, **RET**, RHOH, RNF213, ROS1, RPL22, RPN1, RUNX1T1, SEPT5, SEPT6, SEPT9, SET, SFPQ, SFRS3, SLC45A3, SRGAP3, SS18, SSX1, SSX2, SSX4, STIL, STL, SUZ12, SYK, TAF15, TAL1, TAL2, TCEA1, TCF12, TCL1A, TCL6, TET1, TFE3, TFEB, TFG, TFRC, THRAP3, TLX1, TPM3, TPR, TRIM27, TRIM33, TRIP11, TSHR, TTL, USP6, WHSC1, WHSC1L1, ZBTB16, ZMYM2, ZNF521 | **ATM**, BLM, BMPR1A, **BRCA1, BRCA2,** BRIP1, BUB1B, CBLB, CDC73, CDKN2A, CDKN2C, CHEK2, DDB2, DICER1EP300, **ERCC3, ERCC5**, EXT1, EXT2, FAM123B, FANCC, FANCD2, FANCE, FANCF, FANCG, FAS, **FBXW7**, FH, GATA3, KDM5C, KDM6A, KLF6, MAP2K4, **MEN1,** MLH1, MSH2, MSH6, MUTYH, NBN, NF1, NF2, PHOX2B, PMS1, PRF1, PTCH1, **PTEN,** RB1, RECQL4, SDHAF2, **SDHB, SDHC, SDHD,** SETD2, SMAD4, SMARCB1, SUFU, TET2, TNFAIP3, **TP53**, **TSC1**, VHL, WRN, WT1, XPA, XPC |
